# The C16orf87 protein is a subunit of the MIER corepressor complex controlling embryonic development and cell migration

**DOI:** 10.1038/s41598-026-50740-7

**Published:** 2026-04-30

**Authors:** Tanel Punga, Mårten Larsson, Endrina Mujica, Fabio Rabelo Melo, Christoph Metzendorf, Hanqing Zhang, Dandan Wang, Marcel den Hoed, Leif Andersson, Débora Parrine

**Affiliations:** 1 Department of Medical Biochemistry and Microbiology, Uppsala University, Uppsala, Sweden; 2 Department of Pharmaceutical Biosciences, Uppsala University, Uppsala, Sweden; 3 The Beijer Laboratory, Department of Immunology, Genetics and Pathology, Uppsala University, Uppsala, Sweden; 4 Department of Veterinary Integrative Biosciences, Texas A&M University, College Station, USA; 5 Present Address: National Bioinformatics Infrastructure Sweden (NBIS), Department of Biochemistry and Biophysics, Stockholm University, Stockholm, Sweden

**Keywords:** C16orf87, HDAC1, MIER1, Chromatin, Migration, Embryonic development, Cell biology, Developmental biology, Genetics, Molecular biology

## Abstract

Histone modifications by histone deacetylases (HDACs) are essential for controlling chromatin structure and regulating gene expression. Functionally, HDACs act as enzymatic subunits within diverse multisubunit corepressor complexes, thereby targeting distinct sets of genes. Here, we identify C16orf87 as a previously uncharacterized functional subunit of the MIER corepressor complex that mediates HDAC1 and MIER1 protein interactions. Homozygous knockout of C16orf87 alters chromatin accessibility and reduces cell migration in human cells, and impairs embryonic development of zebrafish. Based on these findings, we propose renaming C16orf87 as HDAC Interacting Protein (HDIP) to reflect its newly revealed function.

**Supplementary Information:**

The online version contains supplementary material available at 10.1038/s41598-026-50740-7.

## Introduction

Chromatin accessibility is a rate-limiting step in gene expression. The chromatin structure must be modified to allow transcription factors and the basal transcription machinery recruitment to the chromosomal DNA. Among different chromatin modifications, much attention has been given to reversible histone acetylation at the lysine residues since they correlate well with gene expression (histone acetylation) and repression (histone deacetylation). Histone acetyltransferases (HATs) and deacetylases (HDACs) are the enzymes that catalyze the attachment and removal of the acetyl group at the histone lysine residues. Notably, HATs and HDACs show different substrate specificities, cofactor requirements, and subunit compositions in mammalian cells^1^. Of the 18 identified mammalian HDACs, 11 are classified as the classical histone deacetylases (HDAC1-HDAC11), whereas the remaining seven (Sirt1-Sirt7) are NAD^+^-dependent sirtuins^1^. The classical HDACs have been further divided into subclasses based on their subcellular localization, functions, and catalytic activities. The best-studied HDACs, HDAC1 and HDAC2, show over 84% sequence identity and act as the catalytic subunits in the same corepressor complexes^2–4^. Biochemical studies have identified at least six HDAC1/HDAC2-containing multisubunit corepressor complexes, including CoREST, NuRD, Sin3, MiDAC, RERE, and MIER^3–5^. Although the subunit compositions of these complexes are known, the exact functions of several individual subunits remain to be defined^5^. Their distinct compositions target corepressor complexes to specific genomic loci, where they deacetylate histones and regulate key biological processes such as transcription, DNA repair, and replication^6^. Not surprisingly, the malfunctioning of HDAC1 and HDAC2 can have severe consequences. For example, HDAC1 knockout mice die prenatally, while HDAC2 knockouts die within a day after birth^7,8^. Overexpression of HDAC1 and HDAC2 has been observed in various cancer types, including hematological, prostate, gastric, lung, colon, colorectal, cervical, and breast cancers^9^. Considering the role of HDACs in hematological cancer development, several HDAC inhibitors have been clinically validated as potential treatments against leukemias and lymphomas^10^. Moving forward, drugs targeting HDAC-containing corepressor complex subunits are warranted as they may induce more specific effect than the present pan-HDAC inhibitors^11^.

Here, we show that a previously uncharacterized protein, encoded by *C16orf87* (human chromosome 16 open reading frame locus 87), is a functional subunit of the MIER corepressor complex mediating HDAC1 and Mesoderm Induction Early Response 1 (MIER1) protein interactions. Notably, the knockout of the *C16orf87* gene in human cells altered chromatin accessibility and reduced cell migration. In zebrafish, *C16orf87* knockout impaired normal embryonic development. We propose to rename *C16orf87* to HDAC Interacting Protein (HDIP) now that its function has been revealed.

## Results

### The C16orf87 protein structure prediction

**Fig. 1:**
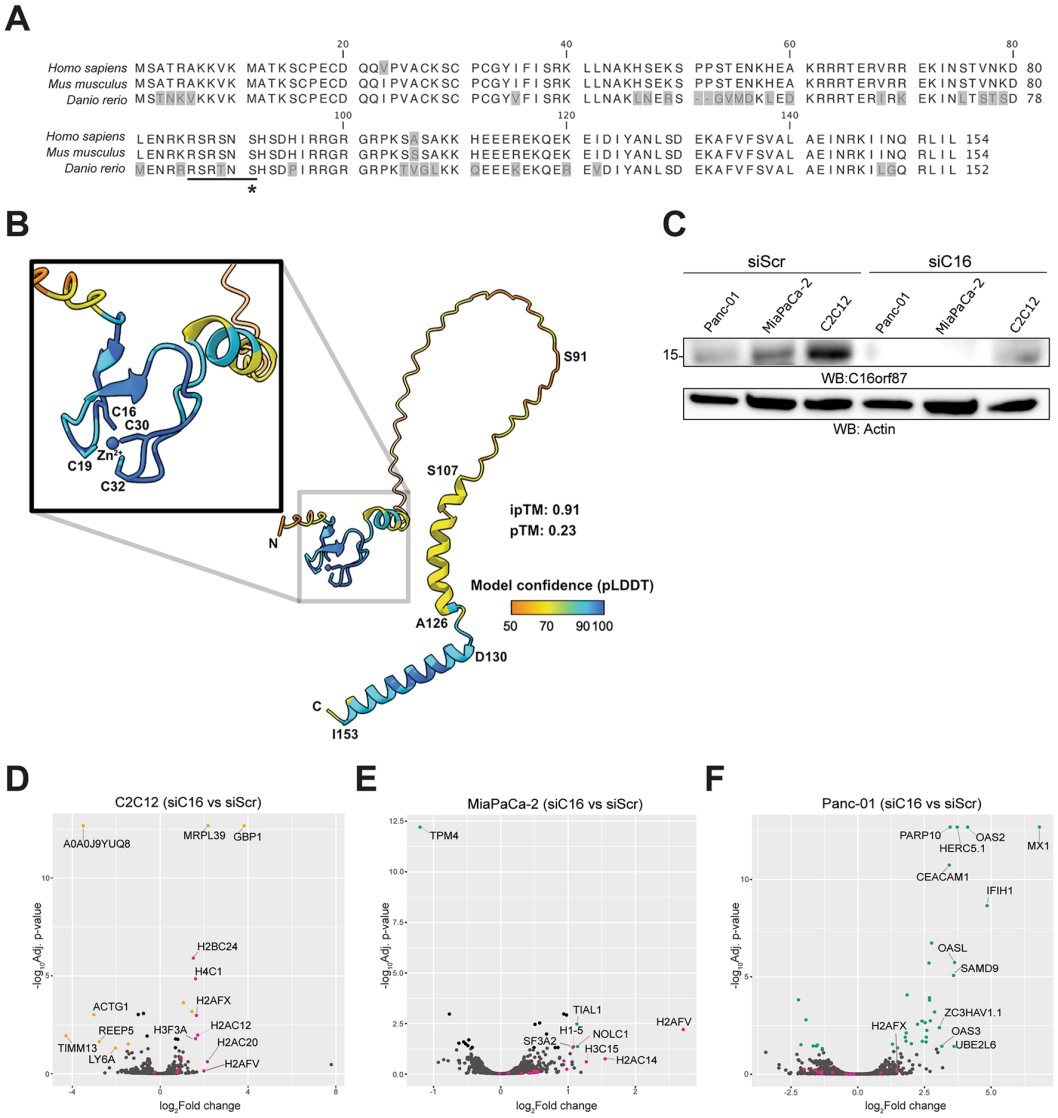
Knockdown of C16orf87 causes minor changes in the host cell protein profile. (**A**) Alignment of human (*Homo sapiens*, UniProtKB accession number Q6PH81), mouse (*Mus musculus*, UniProtKB accession number Q9CR55), and zebrafish (*Danio rerio*, UniProtKB accession number Q6DGQ4) C16orf87 amino acid sequences. Alignment mismatches are indicated in gray boxes. The underlined sequence represents a possible minimal Akt/PKB kinase consensus recognition motif. A Ser91(S91) phosphorylation site is marked with an asterisk. (**B**) Per-residue confidence (pLDDT) coloring of the top-ranked predicted model of C16orf87. In the inset, the predicted zinc-ribbon domain is shown with the zinc-interacting cysteines (Cys16, Cys19, Cys30, and Cys32) indicated around the zinc ion (Zn^2+^). The position of the phosphorylated serine (Ser91), a putative alpha-helix between amino acid residues Ser-107 and Ala-126, and the confidently predicted C-terminal alpha-helix between amino acid residues Asp-130 and Ile-153 are also highlighted. The ipTM and pTM values are annotated. N, N-terminus; C, C-terminus. Figure was rendered using ChimeraX (version 1.8, https://www.rbvi.ucsf.edu/chimerax^62^) (**C**) Western blot (WB) analysis of C16orf87 siRNA (siC16) knockdown in Panc-01, MiaPaCa-2, and C2C12 cell lines. A non-specific, scrambled siRNA (siScr) was used as a control; the WB membrane was probed with the antibodies against C16orf87 and actin. MS-based proteomics analysis of siRNA-treated C2C12 (**D**), MiaPaCa-2 (**E**), and Panc-01 (**F**) cells. Data points corresponding to histones are colored in pink, and statistically significant (*P* < 0.05, fold-change > 1) proteins are colored in yellow (mouse cell line C2C12) and green (human cell lines, Panc-01 and MiaPaCa-2).

Despite multiple studies, the exact functions of several HDAC1- and HDAC2-interacting proteins have remained enigmatic^3,4^. One of these proteins is a small nuclear protein encoded by *C16orf87* on human chromosome 16^3^. Structurally, the C16orf87 protein contains a putative zinc-ribbon domain (ZRD) in its N‐terminus, highlighted by the presence of five cysteine residues (Supplementary Fig. 1a). In addition, human C16orf87 undergoes phosphorylation, with serine 91 (Ser91, S91) as the dominant phosphorylation site (phosphosite.com). The C16orf87 amino acid sequences are highly conserved across human and mouse (99% sequence identity), and there is also a high degree of conservation between the human and zebrafish C16orf87 amino acid sequences (~ 75% sequence identity; Fig. 1a). Human C16orf87 mRNA has a low tissue-specific expression, whereas the encoded C16orf87 protein shows some degree of tissue specificity, as the protein is well detected in the brain and skin tissues (proteinatlas.com).

To predict the three-dimensional structure of C16orf87, we used AlphaFold3^12^. The resulting model is predominantly intrinsically disordered, consistent with its very low predicted template modeling (pTM) score (Fig. 1b, Supplementary Fig. 1b). Near the N-terminus, four out of the five cysteines (Cys16, Cys19, Cys30, and Cys32) are predicted to coordinate a zinc ion (Zn^2+^), forming a zinc-ribbon domain. This domain is modeled with very high confidence (mean predicted local distance difference test (pLDDT) > 90), and therefore, its interaction with Zn^2+^ also results in a very high interface predicted template modeling (ipTM) score. Towards the C-terminus, an alpha-helix spanning Asp130 to Ile153 is modeled with high confidence (mean pLDDT > 90), whereas a second alpha-helix between Ser107 and Ala126 is predicted, although with low confidence. Notably, the UniProtKB entry for C16orf87 (UniProtKB Q6PH81) annotates a putative coiled-coil region between residues 104–132. Because this region has relatively low local confidence (mean pLDDT < 70), we analyzed it using multiple sequence-based coiled-coil prediction tools. Predictions were generated with Waggawagga^13^, which integrates six prediction algorithms (Marcoil^14^, MultiCoil^15^, MultiCoil2^16^, PairCoil^17^, PairCoil2^18^, and NCOILS^19^) for comparative coiled-coil detection. We also used the coiled-coil prediction tools DeepCoil and DeepCoil2^20^ through the MPI Bioinformatics Toolkit^21^ and CoCoNat^22^. Only NCOILS predicted a coiled-coil region (residues 108–131; Supplementary Fig. 1c). However, given the remarkably poor reported performance of NCOILS (Matthews Correlation Coefficient (MCC) of 0.02, which indicates a random prediction, an FDR of 91%, and a false negative rate of 59 to 63%)^23^, and the absence of supporting predictions from the other tools (Supplementary Fig. 1c), we conclude that C16orf87 does not contain a *bona fide* coiled-coil region. The moderate average AlphaFold3 confidence in this segment (mean pLDDT = 64.8) does not strongly support a stable alpha-helix, but intrinsic disorder remains a plausible interpretation. The phosphorylated Ser91 lies within the central intrinsically disordered region of the protein, consistent with a potential regulatory role.

Overall, our analyses suggest that C16orf87 is largely disordered but interrupted by small, ordered modules (a well-defined N-terminal zinc-ribbon domain and a C-terminal alpha-helix). The disordered segments include the Ser91 phosphorylation site and a region previously annotated as a possible coiled-coil (UniProtKB Q6PH81) but not supported by our analyses. This suggests that C16orf87 may act as a flexible interaction scaffold with localized structural elements, which could enable regulatory or interaction-mediated functions.

### Knockdown of C16orf87 causes minor changes in the host cell protein profile

To gain a deeper understanding of C16orf87 function, we used siRNAs (hereafter siC16) to block C16orf87 expression in two human pancreatic cell lines (MiaPaCa-2 and Panc-01) and a mouse skeletal muscle cell line (C2C12), and analyzed protein expression by mass spectrometry (MS). siC16 treatments efficiently reduced C16orf87 protein accumulation in all tested cell lines (Fig. 1c). Subsequent MS-based proteomics analysis comparing siScr- (control, scrambled siRNA) and siC16-treated whole cell lysates indicated that several histone proteins (H4, H3, H2A, and H2B) accumulated in siC16-treated C2C12 and MiaPaCa-2 cell lines (Fig. 1d, e). In contrast, C16orf87 mRNA knockdown in Panc-01 cells was associated with a higher expression of OAS2, IFH1, MX1, HERC5, OASL, SAMD9, and OAS3, proteins known to be involved in the innate immune response, with no significant impact on histone proteins (Fig. 1f). Hence, our data suggest that C16orf87 knockdown caused a significantly higher abundance of histones in MiaPaCa-2 and C2C12 but not in Panc-01 cells.

### CRISPR-Cas9 knockout of C16orf87 reduces Panc-01 cell migration

**Fig. 2:**
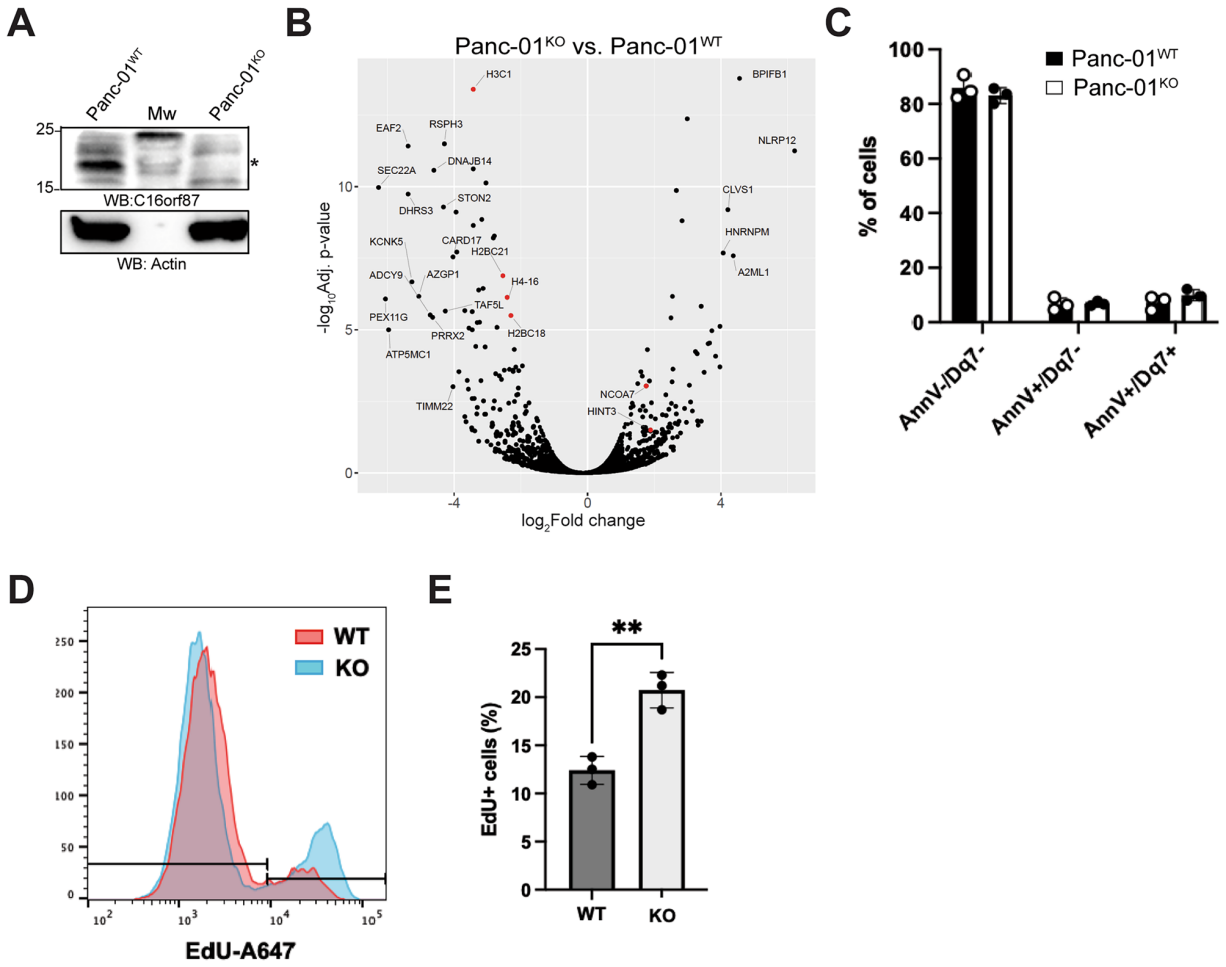
CRISPR-Cas9 knockout of C16orf87 does not affect Panc-01 cell viability. (**A**) Whole-cell lysates of Panc-01^KO^ and Panc-01^WT^ cells were analyzed by western blot (WB), and proteins were detected with the antibodies against C16orf87 and actin. An asterisk indicates the migration of the C16orf87 protein. Mw; molecular weight marker. (**B**) MS-based proteomics analysis of Panc-01^KO^ and Panc-01^WT^ cell lysates (FDR ≤ 0.05, n = 3). Data points representing histones and proteins of interest are highlighted in red. (**C**) Panc-01^KO^ and Panc-01^WT^ cell viability was analyzed by FACS after AnnexinV-FITC (AnnV) and DRAQ7 (Dq7) staining. (**D**) Panc-01^KO^ and Panc-01^WT^ cell proliferation was analyzed by FACS after EdU-A647 incorporation into cells. (**E**) Quantification of the FACS analysis (*P* < 0.01 (**)).

**Fig. 3:**
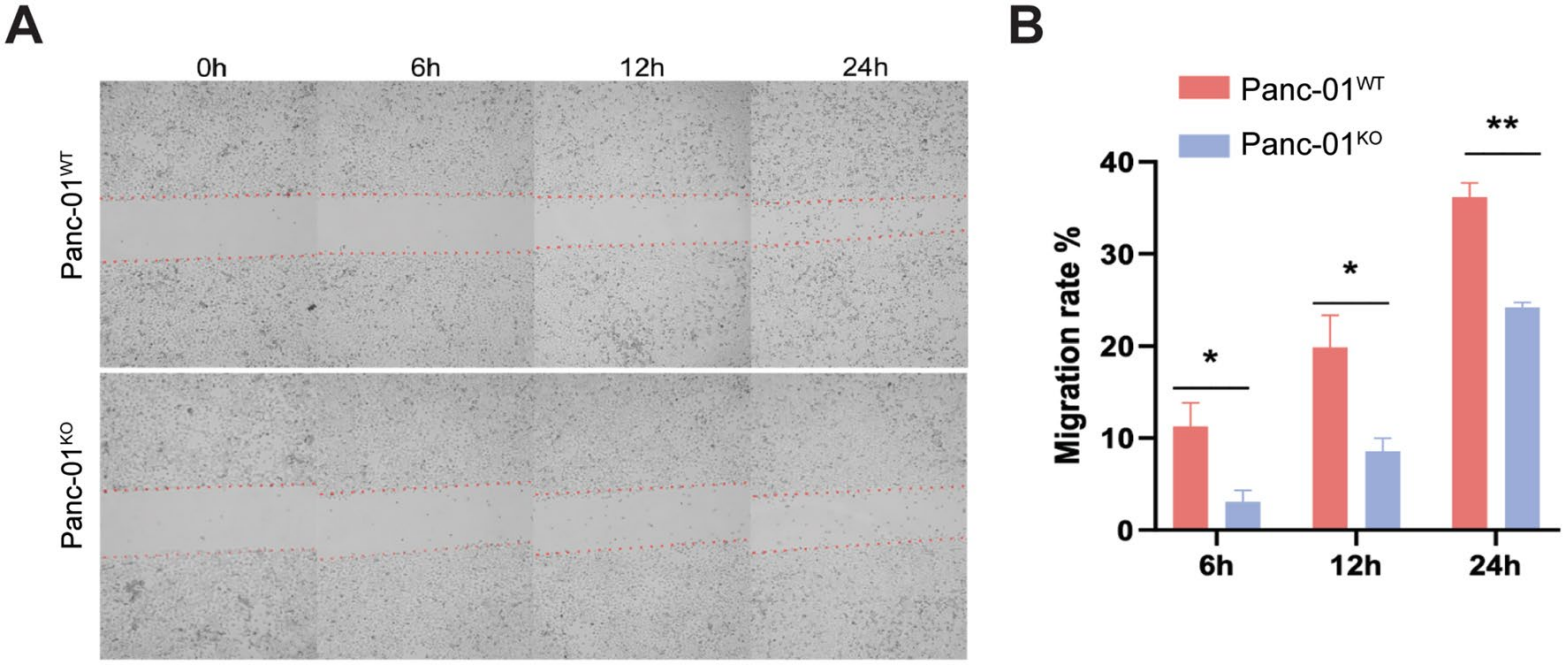
CRISPR-Cas9 knockout of C16orf87 reduces Panc-01 cell migration. (**A**) Microscopy images of the in vitro scratch assay in Panc-01^KO^ and Panc-01^WT^ cells. Images were taken at 0, 6, 12, and 24 h after scratches were applied. (**B**) The cell migration rate was calculated based on the extent of cell coverage within the scratched area (*P* < 0.05 (*) and *P* < 0.01 (**)).

Previous studies have shown that siRNA treatment may confound the interpretation of gene expression data, as small synthetic RNA molecules can elicit unintended innate immune responses^24,25^. As a siRNA-free approach, CRISPR-Cas9 genome editing can mitigate these siRNA-specific side effects. Therefore, we generated a Panc-01 knockout (KO) cell line using CRISPR/Cas9 genome editing. We isolated a cell clone (hereafter Panc-01^KO^) lacking 158 nucleotides in exon 1 of *C16orf87* (Supplementary Fig. 2), consequently interrupting C16orf87 protein expression (Fig. 2a). A subsequent MS-based proteomics analysis between Panc-01^KO^ and Panc-01^WT^ cell lines showed 93 proteins with higher abundance and 116 proteins with lower abundance in the KO cells (log2 fold change threshold = 2, *P* ≤ 0.05, n = 3, Fig. 2b, Supplementary Table 1). Proteins with higher expression in Panc-01^KO^ cells are involved in innate immune response (NLRP12 and BPIFB1), vesicle transport (CLVS1), RNA metabolism (G3BP2 and HNRNPM), cell death (BNIP2), differentiation (BTK), and cellular structure (MYH10) (Supplementary Table 1). Proteins showing lower expression in Panc-01^KO^ cells are involved in transcription regulation (EAF2, TAF5L, and PRPX2), vesicle transport (SEC22A), and peroxisome biogenesis (PEX11G) (Supplementary Table 1). Notably, also the histone proteins H2A, H2B (H2BC21, and H2BC18), H3 (H3C1), and H4 (H4-16) showed reduced accumulation in Panc-01^KO^ cells. Since reduced histone expression can disrupt cell cycle progression and cell viability^26^, we measured cell viability and proliferation rates in Panc-01^KO^ and Panc-01^WT^ cells. Despite the reduced histone abundance, Panc-01^KO^ cell viability was not altered when analyzed by Annexin-V (detects early apoptotic cells) and DRAQ7 (detects late apoptotic cells) staining (Fig. 2c). Surprisingly, Panc-01^KO^ cells showed a significantly increased proliferation rate compared to Panc-01^WT^ cells when analyzed by EdU incorporation during de novo DNA replication (Fig. 2d, e, P = 0.04, n = 3). To further assess whether the absence of the C16orf87 protein alters cell migration, we performed an in vitro scratch assay. This assay measures cell migration by visually analyzing dynamic cell spreading over a predefined time period^27^. In contrast to the elevated EdU incorporation (i.e., read-out for cell proliferation, Fig. 2e), Panc-01^KO^ cells showed a significantly (*P < 0.05, **P < 0.01, n = 3) lower migration rate than the Panc-01^WT^ cells at all tested time points (6, 12, and 24 h; Fig. 3a, b).

Taken together, our data suggest that genomic aberration of the *C16orf87* gene specifically impairs histone protein accumulation, increases cellular proliferation, and reduces cell migration in Panc-01 cells.

### C16orf87 partially mediates HDAC1 and MIER1 protein interactions

**Fig. 4:**
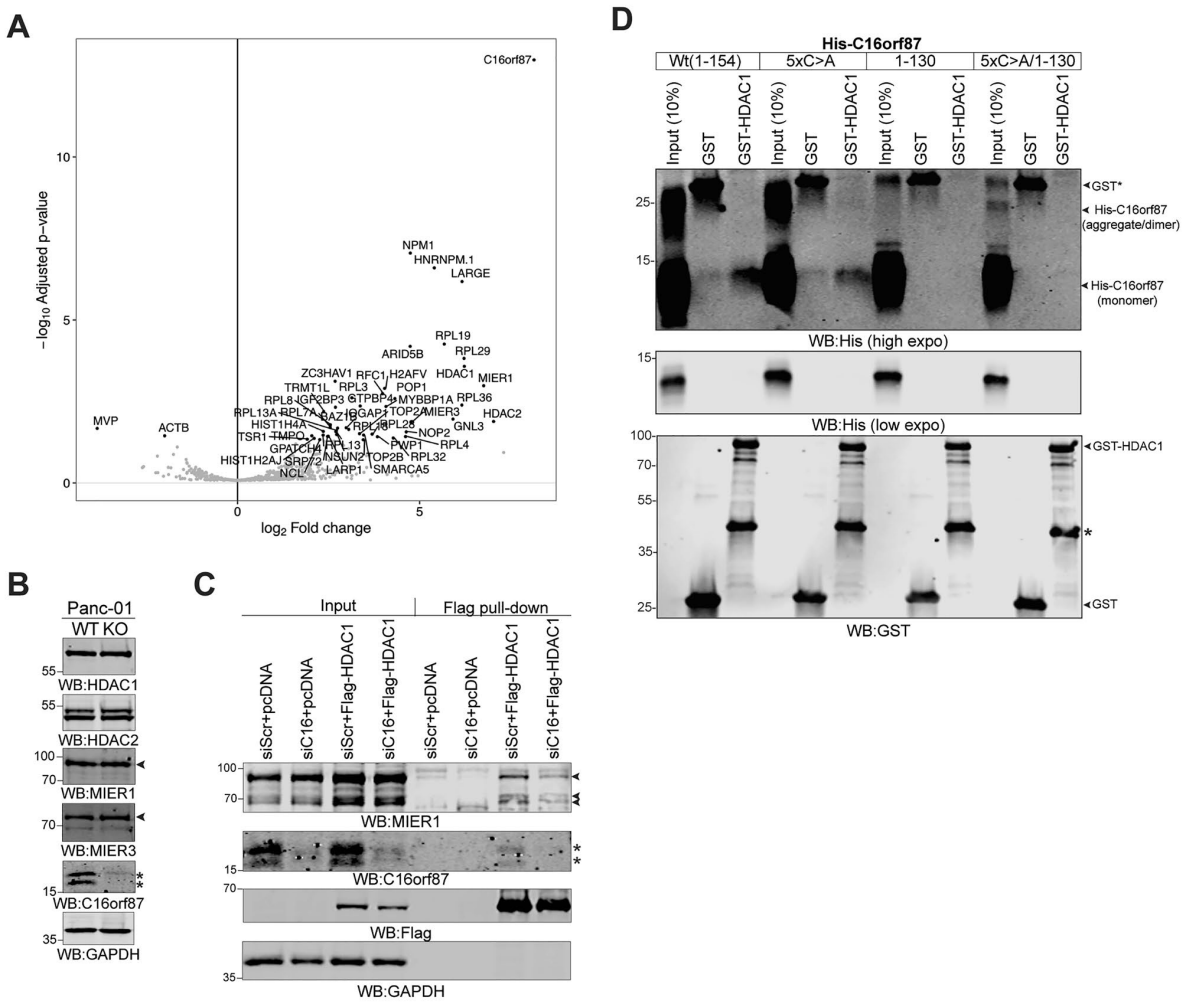
C16orf87 partially mediates HDAC1 and MIER1 protein interactions. (**A**) C16orf87 interacts with the HDAC and MIER proteins. Volcano plot of the IP-MS experiment showing identified proteins interacting with the Flag-C16orf87 protein in HeLa cells. An adjusted *P*-value cut-off of 0.05 and a log2 fold change cut-off of 2 were used. Data are shown from a biological triplicate experiment. (**B**) Lack of C16orf87 does not change HDAC and MIER protein accumulation. Soluble Panc-01^WT^ (WT) and Panc-01^KO^ (KO) whole-cell lysates were analyzed by WB with the indicated antibodies. (**C**) C16orf87 partially mediates HDAC1 and MIER1 interaction. Co-immunoprecipitation of Flag-HDAC1 from siRNA (siC16 and siScr) and pcDNA3-Flag-HDAC1-transfected HeLa cells. Isolated proteins were analyzed by WB with the indicated antibodies. An arrowhead indicates the migration of the MIER1 protein isoforms, whereas an asterisk indicates the migration of the C16orf87 isoforms. (**D**) HDAC1 interacts weakly with C16orf87 in vitro. GST (as a control) and GST-HDAC1 pull-down with bacterially purified 8 × His-tagged C16orf87(Wt, 5 × C > A, 1–130, and 5 × C > A/1–130) proteins. An asterisk indicates a degradation product/partially translated GST-HDAC1. Proteins were detected with the anti-His and anti-GST antibodies.

To identify proteins specifically interacting with C16orf87, we overexpressed a Flag-C16orf87 protein in HeLa cells, followed by Flag-C16orf87 immunoprecipitation and detection of the interacting proteins using an immunoprecipitation-mass spectrometry (IP-MS) analysis. Among the identified proteins that specifically interact with the Flag-C16orf87 protein (Fig. 4a), we focused on those that regulate histone acetylation and chromatin modifications, since the lack of C16orf87 interfered with histone accumulation (Fig. 2b). Notably, we identified that HDAC1 and HDAC2, as well as the transcription factors MIER1 and MIER3, specifically interact with the Flag-C16orf87 protein. Since the HDAC1, HDAC2, and MIER proteins can form a functional MIER corepressor complex^28,29^, we hypothesized that C16orf87 may regulate the composition and/or activity of the complex members. However, the C16orf87 knockout did not alter HDAC1, HDAC2, MIER1, or MIER3 expression levels in Panc-01^KO^ cells (Fig. 4b). To test whether the C16orf87 protein mediates the interaction between different subunits of the MIER complex, we immunopurified Flag-HDAC1-interacting proteins in HeLa cells treated with siRNA against C16orf87. Notably, siRNA treatment reproducibly reduced MIER1 interaction with the Flag-HDAC1 protein (Fig. 4c), suggesting that C16orf87 mediates HDAC1 and MIER1 protein interaction within the MIER corepressor complex.

The C16orf87 protein contains a putative zinc-ribbon domain (ZRD, Fig. 1b), which may mediate protein–protein interactions. However, mutation of five cysteines (C) to alanine (A) (5 × C > A) within the predicted ZRD of C16orf87 did not affect its interaction with HDAC1 and MIER1 (Supplementary Fig. 3a). Since C16orf87 is a phosphoprotein (Supplementary Fig. 1a), we next investigated whether its phosphorylation status influences the interaction of C16orf87 with HDAC1 and MIER1. Available phosphoproteomics studies (phosphosite.org) suggest Ser91 (Supplementary Fig. 1a, S91) as the main C16orf87 phosphorylation site mimicking a minimal Akt/PKB kinase consensus recognition motif (R-X-R-X-X-S/T, Fig. 1a)^30^. To validate Ser91 phosphorylation, we treated C2C12 cells with Leukemia Inhibitory Factor (LIF), a potent cytokine that induces various kinases, including those that phosphorylate and activate Akt/PKB. Indeed, our MS-based phosphoproteomic analysis revealed that LIF treatment led to significantly higher levels of C16orf87 phosphorylation, particularly at Ser91 (Supplementary Fig. 3b). Hence, we tested whether Ser91 phosphorylation was required to maintain the interactions among C16orf87, MIER1, and HDAC1. For this purpose, we generated a Flag-C16orf87 phosphorylation mutant (S91A) and used it along with the wild-type Flag-C16orf87 to isolate MIER1 and HDAC1 proteins. However, both Flag-C16orf87 proteins interacted equally well with the HDAC1, HDAC2, and MIER1 proteins in HeLa cells (Supplementary Fig. 3c). To further characterize the C16orf87-HDAC1 interaction, we performed in vitro protein–protein binding assays using bacterially purified GST-tagged HDAC1 and various His-tagged C16orf87 proteins. In addition to the 5 × C > A mutant, we generated a C16orf87 deletion mutant lacking the C-terminal 24 amino acids (residues 1–130), as well as a combined mutant, C16orf87(5 × C > A/1–130). HDAC1 showed weak binding to the C16orf87 Wt and 5 × C > A mutant, whereas binding was completely abolished for the 1–130 and 5 × C > A/1–130 mutants (Fig. 4d). Notably, His-C16orf87 Wt and the 5 × C > A mutant formed aggregates/oligomers, which were drastically reduced in the 1–130 and 1–5 × C > A/1–130 mutants. We therefore sought to evaluate whether C16orf87 can form a homodimer in the absence of other binding partners. AlphaFold-Multimer^31^ was used to predict the structure of the isolated protein. Although the predicted model displayed a dimeric arrangement mediated by the C-terminal alpha-helices, the associated confidence metrics were very low, with a pTM of 0.18 and an ipTM of 0.09, indicating weak support for a stable intermolecular interaction (Supplementary Fig. 4a, b). However, the ipTM value is known to be affected by extensive intrinsically disordered regions or domains that do not participate in the interaction. A large proportion of C16orf87 is predicted to be disordered (Fig. 1b). To mitigate this issue, alternative metrics have been developed, such as Actual Interface pTM (actifpTM)^32^ or Interaction Prediction Score from Aligned Errors (ipSAE)^33^. Unlike ipTM, they both restrict the analysis to high-confidence interfacial regions and reduce the influence of disordered or non-interacting segments. The calculated values of actifpTM and ipSAE of the C16orf87 dimer were 0.223 and 0.005, respectively, providing no confident support for a homodimerization. These results suggest that the apparent oligomeric arrangement noticed in the western blots most likely reflects limited local contact patches of the hydrophobic C-terminal amino acid residues rather than a biologically meaningful homodimer. Finally, since the C16orf87 protein interacts with HDAC1 (Fig. 4a, c, d), we considered the possibility that C16orf87 may directly influence HDAC1 enzymatic activity. However, incubating the purified C16orf87 protein with purified HDAC1 protein did not alter HDAC1’s enzymatic activity toward the acetylated peptide as the substrate (Supplementary Fig. 5).

Together, our data indicate that the C16orf87 protein acts as a mediator by connecting the HDAC1 and MIER1 proteins, where these interactions occur independently of the C16orf87’s ZRD and Ser91 phosphorylation.

### C16orf87–HDAC1–MIER1 complex structure prediction

**Fig. 5:**
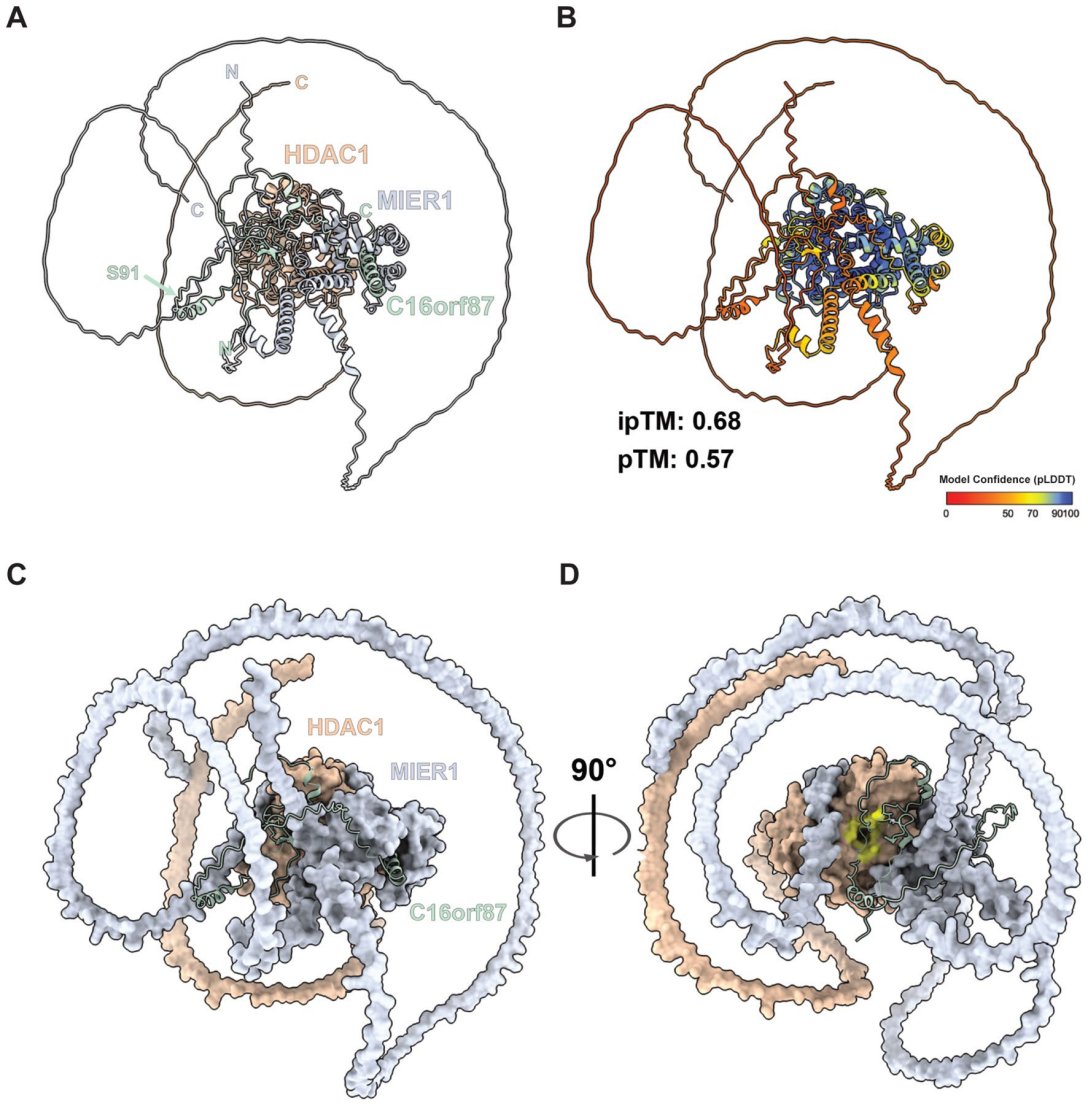
AlphaFold3-predicted model of the C16orf87–HDAC1–MIER1 complex. (**A**) Cartoon representation of the top-ranked predicted complex showing C16orf87 (pastel green), HDAC1 (peach), and MIER1 (lavender grey). The arrow indicates Ser91 in C16orf87. N, N-terminus; C, C-terminus. (**B**) The same model colored by per-residue confidence (pLDDT). Interface predicted TM-score (ipTM) and predicted TM-score (pTM) values for the top-ranked model are shown. (**C**) Surface representation of HDAC1 (peach) and MIER1 (lavender grey) with C16orf87 (pastel green) displayed as a cartoon model. (**D**) View of the model in (C) rotated 90° around the vertical axis. Residues lining the catalytic pocket of HDAC1 are highlighted in yellow. Figures were rendered using ChimeraX (version 1.8, https://www.rbvi.ucsf.edu/chimerax^62^).

We next modeled a possible structure of the C16orf87-HDAC1-MIER1 complex (Fig. 5, Supplementary Fig. 6a–f, Supplementary Video 1). AlphaFold3 predicted a complex with a rigid HDAC1 core and a long, intrinsically disordered C-terminal tail (Supplementary Fig. 6a). MIER1 was largely intrinsically disordered but contained a well-defined central ELM2-SANT module (Supplementary Fig. 6b). The N-terminal ordered portion of the ELM2 domain, which lacks any extensive secondary structure, wraps around the top of HDAC1 with high confidence (mean pLDDT > 80), and the remainder of the ELM2 and SANT domain contacts the side of HDAC1 with similarly high confidence (Supplementary Fig. 6d). The disordered C-terminal region of MIER1 shows only low confidence (mean pLDDT ~ 50) where it approaches the HDAC1 surface and may partially cover the acetate-release channel (Fig. 5c). C16orf87 is predicted to be mostly disordered (Supplementary Fig. 6c), but contains one short, highly confident C-terminal alpha-helix (residues 133–150) that docks between the ELM2 and SANT domains (Fig. 5c, and Supplementary Fig. 6e). The remainder of C16orf87 is flexible and attaches with low confidence (mean pLDDT ~ 50) along the side of HDAC1. The N-terminal zinc-ribbon domain of C16orf87 is predicted near the catalytic channel of HDAC1 but with only moderate confidence (mean pLDDT ~ 70, inter-chain PAE ~ 20 Å) (Fig. 5d).

To examine predicted interfaces, we applied PICKLUSTER^34^, which defines a contact when any heavy atom distance between chains is ≤ 5.0 Å and the local pLDDT value ≥ 50. PICKLUSTER identified three C16orf87–HDAC1 clusters, one C16orf87–MIER1 cluster, and one HDAC1–MIER1 cluster (Supplementary Fig. 7a, Supplementary Tables 2, 3, 4). Cluster 1 of the C16orf87–HDAC1 interface includes the zinc-ribbon domain and also residue Glu98 of HDAC1, which is part of the HDAC1 catalytic pocket. However, the mean local confidence of this region is of low confidence (mean pLDDT ~ 60, mean PAE ~ 21 Å). Clusters 2 and 3 correspond to the C-terminal alpha-helix of C16orf87 docking to the HDAC1 core, both with high confidence (mean pLDDT > 80) and a low PAE (< 3 Å, Supplementary Fig. 7a, Supplementary Table 2). The C16orf87-MIER1 interface cluster involves the C16orf87 C-terminal helix inserted at the junction between the ELM2 and SANT folds (Supplementary Fig. 7a, Supplementary Table 3) with high local confidence (mean pLDDT > 80, PAE < 3 Å). The HDAC1-MIER1 interface highly matches the previously reported ELM2-SANT/HDAC1 structure (Protein Data Bank accession number 4BKX^35^, RMSD 0.285; Supplementary Fig. 8) and is modeled with high confidence (mean pLDDT > 85, PAE ~ 2 Å) (Supplementary Fig. 7a, Supplementary Table 4).

Because the global complex ipTM was modest and inter-chain PAE was elevated, we also evaluated the interfaces using the more stringent software AlphaBridge^36^. The AlphaBridge workflow first merges the pLDDT and PAE metrics into a predicted merged confidence (PMC) matrix (Supplementary Fig. 7b), which is then used to identify multi-component modules within a complex. The predicted distance error (PDE) matrix from AlphaFold3 (Supplementary Fig. 7c) is then masked outside these modules to detect contact links, which are combined to define binary interfaces. From these interfaces, AlphaBridge computes per-residue predicted interaction confidence scores (piCS). The number of detected interfaces depends on the cutoff value applied when masking the PDE matrix. AlphaBridge detected three binary interfaces (Supplementary Fig. 7d, e, Supplemental Table 5) that correspond closely to the PICKLUSTER patches. The C16orf87-HDAC1 interface (matching PICKLUSTER cluster 2) had a piCS score of 0.94, the C16orf87-MIER1 interface (matching the alpha-helix dock) had a piCS score of 0.89, and the HDAC1-MIER1 interface (matching the MIER1 ELM2-SANT/HDAC1 fold) had a piCS score of 0.95. All three interfaces scored 0.99 on the AlphaBridge overall interface confidence metric.

Overall, the model suggests that HDAC1 provides a rigid catalytic core, MIER1 contributes an ordered ELM2-SANT module that docks stably onto HDAC1, and C16orf87, although largely disordered, contributes a confidently placed C-terminal helix at the ELM2-SANT junction and a moderately confident N-terminal zinc-ribbon region near the HDAC1 catalytic channel.

### C16orf87 knockout induces chromatin changes at a subset of genes known to be HDAC targets

**Fig. 6:**
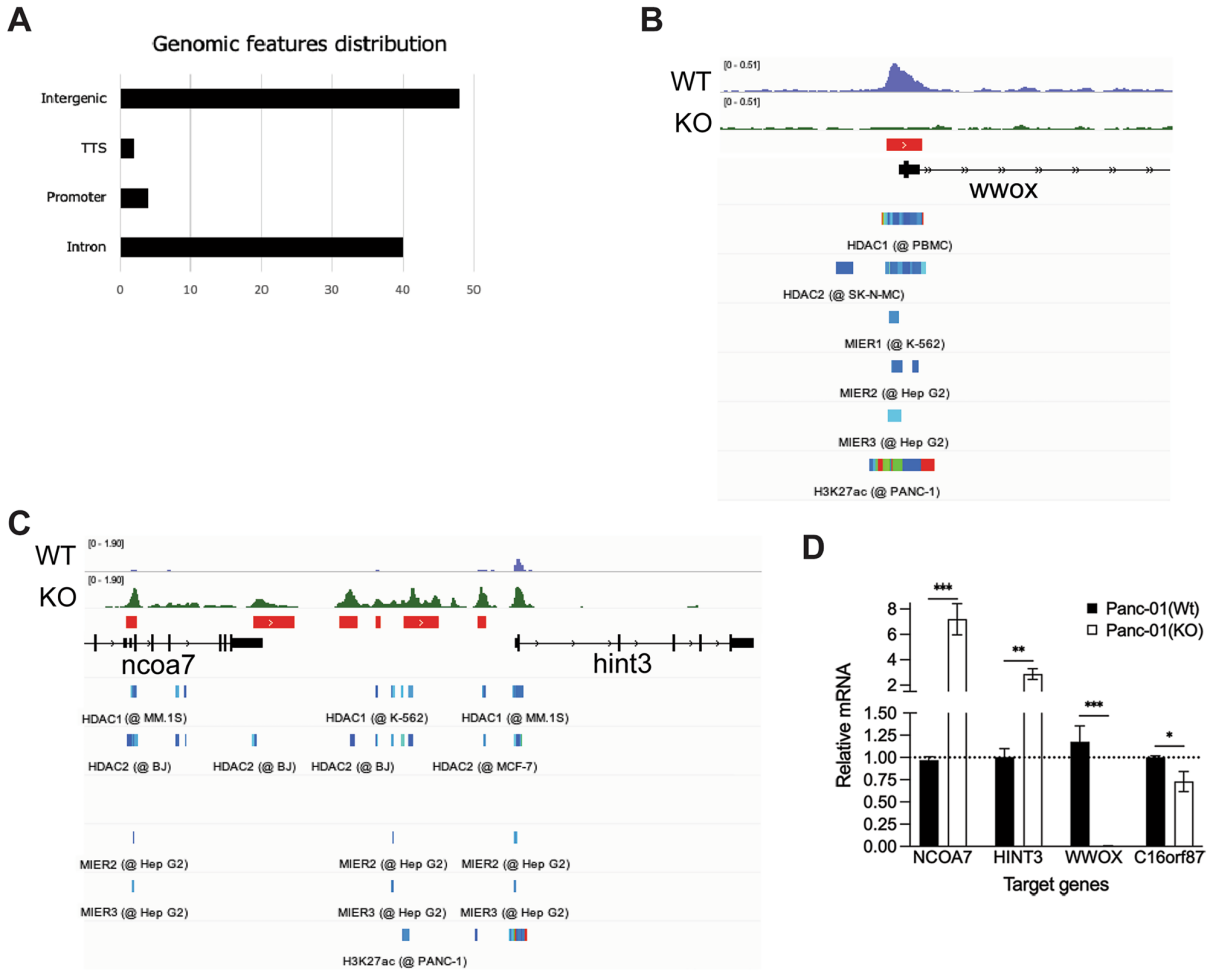
Lack of C16orf87 alters chromatin accessibility. (**A**) Distribution of the more accessible chromatin genomic features identified by ATAC-seq in Panc-01^WT^ and Panc-01^KO^ cells. The X-axis shows values in percentage. Gene locus diagrams showing genomic regions near the *WWOX* (**B**) and *NCOA7-HINT3* (**C**) genes, with peaks representing ATAC-seq reads indicating chromatin accessibility. Data were aligned to available tracks (ChIP-Atlas) of *HDAC1*, *HDAC2*, *MIER1*, *MIER2*, *MIER3*, and *H3K27ac* markers. Panc-01^WT^ and Panc-01^KO^ peaks are shown in blue and green, respectively, and significant differences (FDR threshold: 0.05) in read quantities (peaks), observed in genomic intervals, are shown in red bars on the third track (top to bottom). *WWOX* represents one of the genes with a higher peak on the Panc-01^WT^ compared to Panc-01^KO^. *NCOA7-HINT3* represents one of the genes with higher peaks on the Panc-01^KO^. (**D**) qRT-PCR analysis of the *NCOA7*, *HINT3*, *WWOX*, and *C16orf87* mRNA expression. Relative mRNA expression in Panc-01^WT^ and Panc-01^KO^ cells after normalization to 18S rRNA and considering mRNA levels in Panc-01^WT^ cells as 1.

Since C16orf87 interacts with HDAC1 and HDAC2 (Fig. 4), we hypothesized that the lack of C16orf87 may alter chromatin accessibility. To test this, we performed ATAC-seq (assay for transposase-accessible chromatin using sequencing) of DNA isolated from the Panc-01^WT^ and Panc-01^KO^ cells. Chromatin extracted from Panc-01^WT^ and Panc-01^KO^ cells was sequenced to identify differences in accessible chromatin peaks between the cell lines. A relative quantitation analysis of the results was carried out (n = 3 per cell line, FDR < 0.05). Of the 94 ATAC-seq peaks that differ between Panc-01^WT^ and Panc-01^KO^ cells, two map at the transcription termination site (TTS) and four at promoters/transcription start site (TSS) (Fig. 6a). The remaining are in intergenic regions (48 loci) or introns (40 loci). Knockout of C16orf87 resulted in a significant increase in the number of genomic regions that changed from less accessible to more accessible (78 vs. 16; X_2_ = 40.9 (d.f. = 1), *P* < 0.001). Some ATAC-seq peaks mapped to different parts of the same gene, and the peaks found to have a significant intensity difference between Panc-01^WT^ and Panc-01^KO^ cells mapped to 94 loci within 54 unique genes (Fig. 6b, c, Supplementary Fig. 9a, Supplementary Table 6). In our co-immunoprecipitation assay, MIER1 and MIER3 were identified as C16orf87-interacting proteins (Fig. 4a, c). MIER1 and MIER3, along with MIER2, belong to the *mier* protein family characterized by the presence of the conserved ELM2-SANT domain^37^. Notably, the MIER proteins are regarded as transcription repressor proteins that can recruit HDACs to DNA^29,38^. We therefore examined whether ATAC-seq peaks showing a significant difference in read numbers between Panc-01^WT^ and Panc-01^KO^ cells overlapped with MIER1-3 binding sites detected in previous ChIP-seq datasets (ChIP-Atlas, chip-atlas.org). Peaks aligned to the MIER-binding sites, and particularly to MIER3, were observed in the genes *AKR1E2, ARHGAP18, HINT3, LAMB1, LINC00970, LOC101928051, NUDT7, PALM2-AKAP2, PDE8A, SLC16A9,* and *WWOX* (Supplementary Fig. 9a, Supplementary Table 6). Two genomic regions (*WWOX* and *NCOA7*-*HINT3)* were selected as representative loci that showed clear differences in ATAC-seq read numbers between Panc-01^WT^ and Panc-01^KO^ cells. As shown in Fig. 6b, c, chromatin accessibility at the *WWOX* locus was decreased, whereas accessibility at the *NCOA7*-*HINT3* intergenic region was increased in Panc-01^KO^ cells. Notably, that increased accessibility at the *NCOA7-HINT3* intergenic region (Fig. 6c) correlates with enhanced accumulation of the *NCOA7* and *HINT3* transcripts in Panc-01^KO^ cells (Fig. 6d). In contrast, *WWOX* mRNA showed a drastic reduction in Panc-01^KO^ cells (Fig. 6d), hence correlating with our observed chromatin compaction at the *WWOX* gene by ATAC-seq (Fig. 6b). Additionally, two CRISPR/Cas9-edited Panc-01 clones (H5 and H9) were analyzed for the mRNA alterations originally observed in clone A6 (referred to as Panc-01^KO^ throughout the manuscript) (Supplementary Fig. 10a). Although clones H5 and H9 showed residual C16orf87 expression (Supplementary Fig. 10b), due to a heterozygous knockout, expression of *NCOA7* and *HINT3* were elevated similar to the A6 knockout clone. Notably, *WWOX* expression in clones H5 and H9 was not as drastically affected as in clone A6. This differential response suggests a dose-dependent, locus-specific regulatory role for the C16orf87-HDAC1-MIER1 complex. Reduced C16orf87 levels are associated with increased expression of *NCOA7* and *HINT3*, consistent with transcriptional repression of these genes at physiological C16orf87 levels. In contrast, near-complete elimination of C16orf87 expression (as in A6 clone) is associated with a drastic reduction in *WWOX* gene expression, whereas partial C16orf87 levels do not appear sufficient to produce this effect. These observations suggest that physiological levels of C16orf87 may be required to maintain or promote *WWOX* expression, although the underlying mechanism remains unclear. Consistent with the CRISPR/Cas9 knockout data, *NCOA7* and *HINT3* mRNA levels were also elevated in siC16-treated HeLa cells (Supplementary Fig. 10c). We also assessed whether the differential peaks overlapped with the binding sites for HDAC1 and/or HDAC2, and found alignment with 24 and 25 peaks, respectively. Ten peaks overlapped with the histone H3 lysine 27 acetylation (H3K27ac) mark, a well-established feature of active enhancers^39^, at loci including *ITGB6, LOC100130298, SLC9A7, ARHGAP18, LAMB1, HINT3, PDE8A, WWOX, NUDT7, ZNF706* (Fig. 6b, c, Supplementary Fig. 9a).

Overall, disruption of the *C16orf87* gene altered specific chromatin accessibility patterns and increased the number of accessible loci in Panc-01 cells. Many of the genes showing altered accessibility were previously reported targets of HDAC1 and/or HDAC2, and several overlapped with known MIER protein-binding sites.

### C16orf87 function is essential for normal embryonic development in zebrafish

**Fig. 7:**
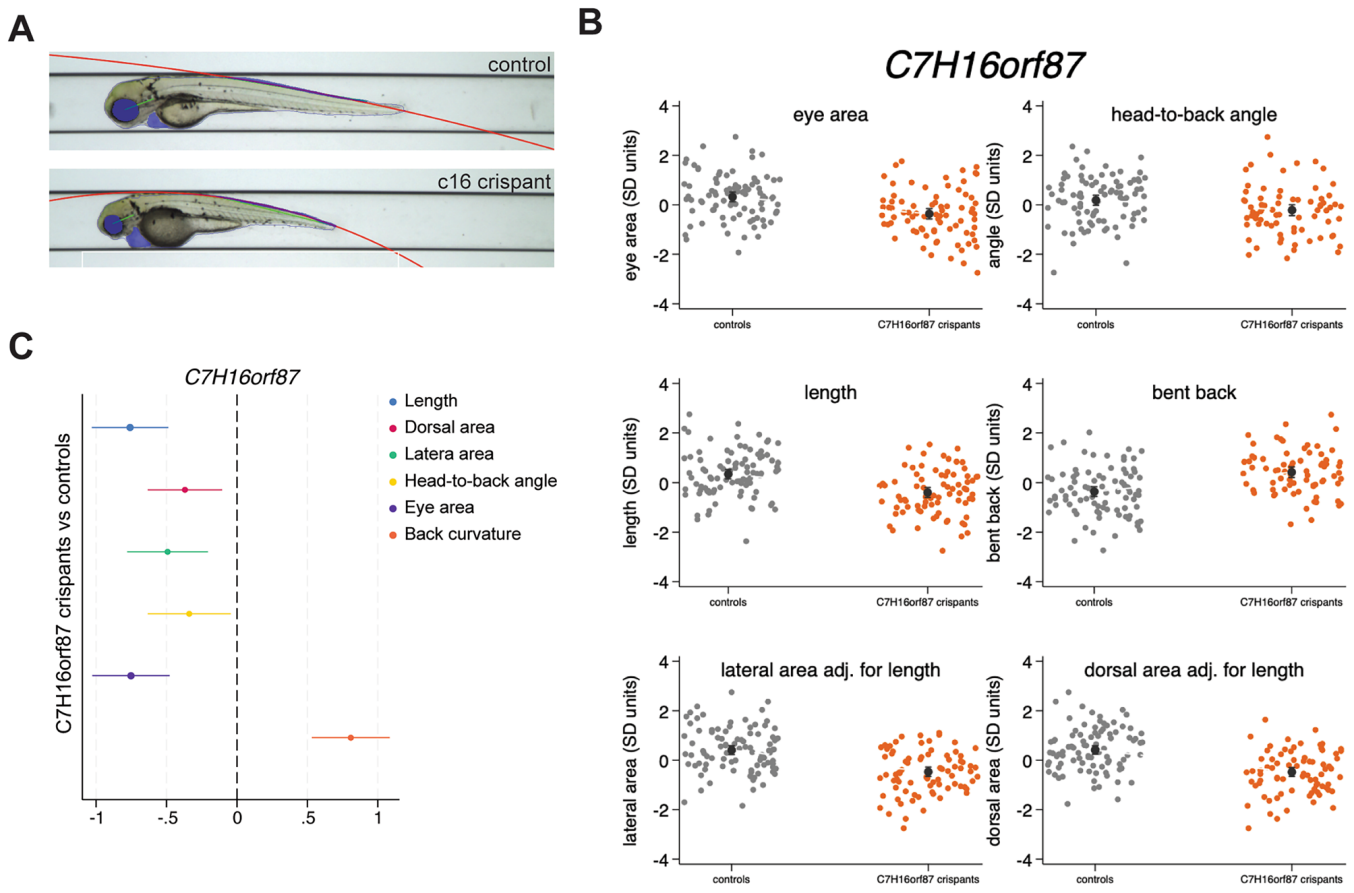
C16orf87 deficiency affects the embryonic development of zebrafish. (**A**) Lateral view of a *C7H16orf87* (human *C16orf87* ortholog) crispant larvae versus its sibling control, highlighting some developmental traits examined: eye (blue), body surface area (blue lines/contour), head-to-back angle (green lines), and curvature of the back (red curve). (**B**) Margins and scatter plots of the most representative developmental traits measured (eye area, head-to-back to angle, length, back curvature) in crispants (orange) and controls (grey) are adjusted for time of imaging. Each orange/grey dot is a larva. (**C**) Forest plot of effect size (dot) and confidence intervals (lines) for the effect of mutations in *C7H16orf87* vs. sibling controls on developmental traits expressed in SD units. Linear regression analyses were adjusted for time of imaging. Zero (red line) corresponds to no difference between crispants and controls.

MIER family proteins contribute to organismal development and differentiation through their interactions with HDACs^38,40^. To assess the role of C16orf87 in embryonic development, we used CRISPR-Cas9 to generate zebrafish crispants with presumed loss of *C7H16orf87* function, i.e., the zebrafish orthologue of the human *C16orf87*. The phenotypes of crispants targeted at *C7H16orf87* and *kita* (control gene) were compared with siblings targeted only at *kita* (n total = 168, where 90 are sibling controls and 78 are crispants). Several developmental parameters were measured at 72 h post-fertilization: body length, dorsal and lateral area, eye area, head-to-back angle, and back curvature (Fig. 7a)^41^. Linear regression analyses adjusted for time of day at imaging showed that C7H16orf87 crispants have impaired early development with crispants being shorter (β ± SE in SD units; − 0.74 ± 0.14, *P* = 3.2 × 10 ^−7^) with smaller eyes (− 0.68 ± 0.14, *P* = 4.8 × 10^−6^), a smaller angle between the head and back (− 0.40 ± 0.15, *P* = 8.6 × 10^−3^), and a more pronounced back curvature (0.79 ± 0.15, *P* = 2.8 × 10^−7^) (Fig. 7b). Lateral and dorsal areas, additionally adjusted for body length, were also affected by the absence of functional *C7H16orf87* (− 0.30 ± 0.08, *P* = 1.3 × 10^−4^ and − 0.42 ± 0.09, *P* = 1.02 × 10^−5^, respectively; Fig. 7b, c). Hence, our analysis of the C16orf87 orthologue knockout in zebrafish revealed significant developmental defects affecting multiple body structures.

## Discussion

Despite the first report of the human genome sequence more than two decades ago^42^, we still lack a basic understanding of the role of many genes. Many of these uncharacterized genes encode enigmatic proteins and are named by their chromosomal location rather than their function^43^. One example is *C16orf87*, a gene on human chromosome 16 that encodes an uncharacterized protein. The present study provides biochemical and functional characterization of this protein. We propose renaming C16orf87 to HDAC1 Interacting Protein (HDIP) now that some of its functions have been revealed.

Our proteomics and biochemical characterizations showed that C16orf87 specifically copurified with the HDAC1, HDAC2, MIER1, and MIER3 proteins (Fig. 4a and Supplementary Fig. 3c), previously identified as subunits of the MIER corepressor complex^29,44^. Based on these studies, MIER1 is regarded as the main scaffold protein of the complex, recruiting the catalytic subunits (HDAC1 and HDAC2) to the chromatin to block gene expression^28,29,44,45^. Interestingly, MIER1 can interact directly with histones H2A and H2B, as well as the intact histone octamer. This unusual binding mechanism may deliver HDAC1 and HDAC2 directly to the nucleosomes along with another heterochromatin-forming protein, BAHD1^29^. Our results show that the absence of the C16orf87 protein does not alter the expression of HDAC1, HDAC2, MIER1, or MIER3 (Fig. 4b), suggesting that C16orf87 does not regulate the stability of the individual subunits in Panc-01 cells. Instead, we think that C16orf87 acts as a mediator between the catalytic (HDAC1) and scaffold (MIER1) subunits, as the lack of C16orf87 reduced MIER1 and HDAC1 interaction (Fig. 4c). The observation that C16orf87 interacts with HDAC1 (Fig. 4a, Supplementary Fig. 3a, c) but does not alter its enzymatic activity (Supplementary Fig. 5) in vitro further supports a role of the C16orf87 protein as a mediator within the MIER corepressor complex and not as an HDAC1 cofactor. Our MS analysis did not reveal significant enrichment of nucleosomal histones or BAHD1 as interacting proteins of C16orf87. These data suggest that C16orf87 is required only for the assembly of the HDAC1-MIER1 protein subcomplex and not for maintaining the MIER complex containing BAHD1. We also detected MIER3 as an interactor of C16orf87 (Fig. 4a). Since MIER3 shows a very weak interaction with HDAC1 and HDAC2^28^, we focused only on validating MIER1-HDAC1 interactions. Notably, the lack of C16orf87 reduced the accumulation of histones H2A and H2B in Panc-01 cells, as determined by our MS-based analysis (Fig. 2b). One possible explanation is that reduced formation of the MIER1-HDAC1 complex in C16orf87-deficient cells (Fig. 4c) leads to decreased levels of soluble histones H2A and H2B. Although hypothetical, the recent finding that MIER1 binds histones H2A and H2B with high affinity^29^ supports a model in which the regulation of H2A and H2B protein levels involves the MIER1-C16orf87-HDAC1 protein complex. It is important to mention that the MS data from siRNA-treated cells (Fig. 1d–f) and CRISPR-Cas9-edited cells (Fig. 2b) showed minimal overlap. This likely reflects the transient nature of siRNA knockdown versus the permanent disruption by CRISPR–Cas9 and their distinct off-target/compensatory effects. Nevertheless, since our functional readouts (cell migration, chromatin accessibility) were obtained in CRISPR-Cas9-modified cells, we believe that the protein expression changes in Panc-01^KO^ cells (Fig. 2b) underlie these phenotypes.

Structurally, C16orf87 contains a putative zinc-ribbon domain (ZRD) encompassing five conserved cysteine residues (Fig. 1a, b) and a dominant phosphorylation site (S91). However, mutating these residues did not change C16orf87 binding to HDAC1 and MIER1 in a HeLa cell line (Fig. 4d, Supplementary Fig. 3a, c). Therefore, the integrity of the ZRD and residue S91 appears dispensable for the mediator function of C16orf87 within the MIER complex. This suggests that these structural characteristics (ZRD) and modifications (Ser91 phosphorylation) are necessary for yet-uncharacterized functions of the C16orf87 protein. In future studies, it will be important to investigate the role of the S91 phosphorylation, perhaps in cellular differentiation, as it conforms to the minimal consensus recognition motif of the PKB/Akt kinase^30^ (Fig. 1a) and is highly enriched in mouse cells treated with LIF, a known cellular differentiation halting factor^46^ (Supplementary Fig. 3b). By applying the ATAC-seq approach, a genome-wide method that identifies open chromatin regions, we observed a significantly higher number of accessible chromatin regions in Panc-01^KO^ compared to Panc-01^WT^ cells. Since open chromatin correlates with the accumulation of acetylated nucleosomal histones, our data suggest that recruitment of the HDAC-containing corepressor complexes to DNA is reduced in Panc-01^KO^ cells. Notably, the increased chromatin accessibility at the *NCOA7* and at the intergenic region between the *NCOA7* and *HINT3* genes (Fig. 6c) correlates with increased accumulation of NCOA7 and HINT3 mRNAs and proteins in Panc-01^KO^ cells (Figs. 2b, 6d, Supplementary Fig. 10a, c, Supplementary Table 6). The *NCOA7–HINT3* intergenic region has been identified as a bidirectionally transcribed CAGE enhancer by the FANTOM5 consortium^47^. This suggests that the C16orf87 protein specifically controls chromatin accessibility at the *NCOA7–HINT3* intergenic enhancer, thereby regulating the expression of both the upstream gene (*NCOA7*) and the downstream gene (*HINT3*). The correlation between open chromatin and increased protein accumulation is not universal in Panc-01^KO^ cells. For example, some genes (*LAMB1 and ARHGAP18*) exhibit increased chromatin accessibility within intronic regions but reduced protein expression in Panc-01^KO^ cells (Supplementary Fig. 9a, Supplementary Table 1, 6), which can be explained by the binding of transcription repressor factors. Collectively, these observations suggest that C16orf87 is involved in chromatin compaction at specific genomic regions, such as intergenic enhancer sequences, rather than at generic HDAC targets, to repress the expression of target genes.

Based on our cell viability and proliferation assays, the lack of C16orf87 does not cause apoptotic cell death but instead enhances cell proliferation (Fig. 2d, e). Our cell proliferation assay measures EdU-A647 (5-ethynyl-2´-deoxyuridine) nucleotide incorporation during de novo DNA replication^48^. Notably, histone acetylation enhances the recruitment of DNA replication factors to the replication origin^49^. Hence, the improved EdU-A647 incorporation in Panc-01^KO^ cells may be due to more accessible, acetylated chromatin. To overcome the limitations of the EdU-A647 incorporation assay, we performed the scratch assay, which tracks cell migration over a longer period than EdU incorporation. Interestingly, the lack of C16orf87 slowed down cell migration, suggesting that the protein is needed for efficient cell growth (Fig. 3). The importance of C16orf87 in cell growth was further supported by zebrafish experiments, where disruption of the C7H16orf87 locus in crispants affected embryonic development (Fig. 7). The zebrafish C7H16orf87 protein shows 76% amino acid sequence homology with the human C16orf87 protein (Fig. 1a), suggestive of similarity in function. Like human cell lines, the zebrafish crispants for *C7H16orf87* are viable but show developmental abnormalities. The severe impact on early-stage zebrafish development demonstrates the critical role of C7H16orf87 during embryonic development, even though the ablation of C7H16orf87 did not cause early mortality. Hence, C16orf87 may have a conserved role in organismal development, although it does not appear to be essential.

During the final stages of our manuscript preparation, a study by Nde and colleagues^50^ reported some of the structural characteristics of the C16orf87 protein. Consistent with our findings, they demonstrated that C16orf87 forms a stable complex with HDAC1, HDAC2, and MIER1. Furthermore, in agreement with our results, the authors found that C16orf87 does not influence HDAC enzymatic activity. However, in contrast to our study, their work did not investigate the functional consequences of C16orf87 expression and ablation in either a cellular or in vivo model. Collectively, our findings reveal novel functions of C16orf87 and suggest that future studies should elucidate the molecular mechanisms by which this protein contributes to the regulation of cell growth and embryogenesis.

## Methods

### Cell culture

Human pancreatic cancer cell lines Panc-01 (ATCC, CRL-1469) and MiaPaCa-2 (ATCC, CRL-1420), mouse skeletal muscle cell line C2C12 (ATCC, CRL-1772), and human cervical cancer cell line HeLa S3 (ATCC, CCL-2.2) were used in this study. The HeLa S3 cells were grown in Dulbecco’s Modified Eagle’s Medium (DMEM, Thermo Fisher Scientific) supplemented with 10% (v/v) fetal bovine serum (FBS, Thermo Fisher Scientific) and penicillin–streptomycin solution (PEST, Thermo Fisher Scientific). The other cell lines were grown in DMEM (Gibco) with added L-glutamine, supplemented with 10% (v/v) heat-inactivated fetal bovine serum (Gibco) and penicillin/streptomycin/L-glutamine (Pen Strep, Gibco) at 0.2 µg/mL, 0.2 U/mL, and 0.2 µg/mL, respectively. The cells were maintained in a humidified incubator at 37ºC and 5% CO_2_.

### Plasmid DNA and siRNA transfections

The mouse C16orf87 DNA sequence was synthesized and cloned into the pcDNA3.1 + N-DYK vector (GenScript) to express an N-terminal Flag-tagged C16orf87 fusion protein. The pcDNA3-Flag-HDAC1 plasmid was generated by inserting human HDAC1 cDNA^51^ into the pcDNA3(N-Flag) vector, whereas an empty pcDNA3(N-Flag)^52^ plasmid was used as a control in transfection experiments. The mouse C16orf87 (Wt), mutants 5 × C > A (point mutation of Cys16Ala, Cys19Ala, Cys27Ala, Cys30Ala, and Cys32Ala), 1–130 (deletion of C-terminal 24 amino acids), and 5 × C > A/1–130 (combination of the 5 × C > A and 1–130 mutants) were cloned into the pSY5 vector to express 8 × His-C16orf87 fusion proteins^53^. Plasmids encoding GST and GST-HDAC1 have been described previously^51^. All plasmid transfections were done using jetPRIME transfection reagent (Polyplus) following the manufacturer’s guidelines. To eliminate C16orf87 expression, Silencer Select siRNA against human C16orf87 (siC16, Thermo Fisher Scientific, s52076) was used. Silencer Select Negative Control #1 siRNA (siScr, Thermo Fisher Scientific, 4390843) was used as a control. The siRNAs were transfected using the jetPRIME transfection reagent (45 nM) for 48 h. For some of the experiments (Fig. 1d–f), the Trifecta siRNA kits targeting human and mouse C16orf87 were purchased from IDT Technologies and transfected using Lipofectamine 3000 reagent (Thermo Fisher Scientific) using 10 nM as the final concentration for 48 h.

### Genome editing (CRISPR-Cas9) of Panc-01 cells

Panc-01 cells were genetically engineered using the CRISPR-Cas9 system. gRNAs were designed using the CHOPCHOP tool (v3)^54^. The ribonucleoprotein (RNP)-mediated method was applied by constructing a complex containing the crRNA (5′-CCACCTGTTGGTCGCACTCGGGG-3′), tracrRNA-ATTO550, and the spCas9 protein (IDT Technologies). The RNP complex was transfected using the JetCRISPR transfection agent (Polyplus). The cell population with a high ATTO550 signal (≥ 20%) was sorted into single-cell suspensions in a 96-well plate using the FACS Aria II cell sorting system (BD Biosciences). Single cells were grown into colonies, followed by PCR screening and sequencing of the C16orf87 gene locus (primer sequences are available in the Supplementary Materials and Methods). Throughout the manuscript, the C16orf87 knockout clone A6 has been used (referred to as Panc-01^KO^).

### SDS-PAGE and western blotting

Panc-01 cell pellets were lysed in RIPA buffer, as described previously^55^. Soluble proteins were separated on homemade 12% or 15% SDS-PAGE, transferred to nitrocellulose filter and incubated with the antibodies against MIER1 (Proteintech, 11452-1-AP), MIER3 (Proteintech, 17543-1-AP), HDAC1 (Thermo Fisher Scientific, PA1-41120), HDAC2 (Proteintech, 12922-3-AP), C16orf87 (Thermo Fisher Scientific, PA5-98546), GAPDH (Proteintech, 60004-1-Ig), GST (Cytiva, 27-4577-01), His (Thermo Fisher Scientific, MA1-21315), α-tubulin (Santa Cruz, sc-69969), or actin (Sigma-Aldrich). Proteins were detected with fluorescence-labeled secondary antibodies (IRDye, LI-COR) and visualized with the Odyssey CLX imaging system (LI-COR). Alternatively, proteins were detected using HRP-conjugated secondary antibodies in the presence of the ECL detection reagents (BioRad). For some of the experiments (Figs. 1 and 2), cells were lysed in M-PER (Thermo Fisher Scientific) buffer supplemented with Halt protease inhibitor cocktail (Thermo Fisher Scientific) and separated on commercial Any-Kd SDS-PAGE (BioRad).

### Co-immunoprecipitation

HeLa cells grown on 100 mm (Flag-C16orf87) or 6-well (Flag-HDAC1) tissue culture plates were transfected with the pcDNA3(N-Flag), pcDNA3.1 + N-DYK-C16orf87, or pcDNA3-Flag-HDAC1 plasmids. At about 24 h post-transfection, cells were lysed in Pierce IP Lysis Buffer (Thermo Fisher Scientific) supplemented with Halt protease inhibitor cocktail (Thermo Fisher Scientific) for 30 min on ice. The soluble cell lysate was immunoprecipitated overnight at 4 °C with anti-Flag (M2) magnetic beads (Merck). The beads were washed 4 × 0.5 mL in lysis buffer. The Flag-C16orf87 samples were separated on 12% SDS-PAGE and analyzed by western blotting as above. The Flag-HDAC1 protein was detected with an anti-Flag (Proteintech, 20543-1-AP) antibody.

### His-C16orf87 and GST-HDAC1 protein purification

Plasmids encoding different 8 × His-C16orf87, GST, and GST-HDAC1 proteins were transformed into BL21(DE3) competent cells, bacterial cultures were grown to OD_600_ = 0.5, and protein expression was induced with 1 mM IPTG at 37 °C for 3 h. Soluble His-tagged proteins were purified with Ni–NTA (Qiagen) as previously^56^, and the eluted proteins were dialyzed against buffer H (20 mM Tris–HCl, pH 8.0, 100 mM NaCl, 10% glycerol, and 1 mM MgCl_2_). Dialyzed soluble proteins were stored at -80 °C. GST and GST-HDAC1 proteins were purified using Glutathione Sepharose 4B (Cytiva) as described previously^51^.

### GST-HDAC1 pull-down

Approximately 1 µg of GST or GST-HDAC1 protein bound to Glutathione Sepharose 4B beads was incubated with 1 µg of purified His-C16orf87 protein in binding buffer (50 mM Tris–HCl, pH 7.5, 100 mM NaCl, 10% glycerol, 0.5% NP-40, and 10 mM MgCl₂). After 1 h of incubation at 4 °C with end-over-end rotation, the beads were washed 4 × 1 mL with binding buffer. Proteins were eluted in 2 × SDS loading dye and analyzed by SDS-PAGE on Any kD gels (BioRad).

### Mass spectrometry

To obtain whole cell samples for the MS analysis (Figs. 1d–f, and 2b), cell pellets were extracted using M-PER extraction buffer (Thermo Fisher Scientific) and quantified using the Pierce BCA Protein Assay (Thermo Fisher Scientific). Immunoprecipitated Flag-C16orf87 interacting proteins (Fig. 4a) were isolated as described above. Protein lysates were separated on a 4–15% gradient SDS-PAGE, and subsequently stained using a QC colloidal Coomassie stain (BioRad). Gel lanes were cut into 2–10 pieces (fractions) and reduced in-gel with 10 mM DTT, alkylated with 55 mM iodoacetamide, and digested overnight with 17 ng/µL sequencing-grade trypsin (Promega) at 37 °C using a slightly modified in-gel digestion method^57^ . The generated peptides were eluted from the gel pieces with 1% (v/v) formic acid (FA) in 60% (v/v) acetonitrile, dried down by vacuum centrifugation, and finally dissolved in 1% (v/v) FA. Peptides were desalted using StageTips (Thermo Fisher Scientific), dissolved in solvent A (0.1% (v/v) FA), and resolved on a 75 µm × 50 cm, 2 µm particle size, EASY-Spray PepMap RSLC C18 column (Thermo Fisher Scientific) heated to 45 °C, and coupled to a 75 µm × 2 cm, 3 µm particle size, Acclaim PepMap 100 pre-column (Thermo Fisher Scientific) run on an EASY-nLC 1000 UHPLC (Thermo Fisher Scientific). A gradient of 2–40% of solvent B (0.1% (v/v) FA in acetonitrile) was run at 250 nL/min for 150 min, and the eluted peptides were injected into an Orbitrap Fusion Tribrid mass spectrometer (Thermo Fisher Scientific) run at Top Speed (max cycle time 3 s) in data-dependent acquisition mode. The ion-transfer tube temperature was set at 275 °C, and the spray voltage was set to 2.4 kV. Full scan spectra (m/z 400–2000) at a resolution of 120,000 at m/z 200 were analyzed in the Orbitrap and collected in profile mode. The automatic gain control (AGC) target was set to 2.0 × 10^5^ with a maximum injection time of 100 ms. Peptides with an intensity of above 5.0 × 10^3^ were selected for collision-induced dissociation fragmentation at a collision energy of 30%. The peptide fragments were analyzed in the linear ion trap at centroid mode with an AGC target of 1.0 × 10^4^ and a maximum injection time of 40 ms. A dynamic exclusion period was set at 1 min.

Raw data were analyzed using the MaxQuant quantitative proteomics software package (version 2.1.0.0)^58^ or for Panc-01^KO^ vs Panc-01^WT^ using Proteome Discoverer (version 2.5, Thermo Fisher Scientific). For MaxQuant, carbamidomethylation of cysteines was set as a fixed modification, and methionine oxidation and protein N-terminal acetylation as variable modifications. MS1 Orbitrap tolerance was set to 20 ppm and MS2 iontrap tolerance to 0.5 Da, and match-between-runs was enabled. The search was made against the UniProtKB/SwissProt *Homo sapiens* proteome (UP000005640, 2022-04-28, Panc-01, MiaPaCa-2, and HeLa S3), *Mus musculus* proteome (UP000000589, 2022-12-01, C2C12), and the MaxQuant potential contaminants databases. A maximum of two miscleavages per peptide was allowed. A decoy search was performed against the reversed databases, with both peptide and protein false discovery rates set to 1%. The output was filtered by removing hits from the reversed databases, proteins identified only by site (only from a modification), and potential contaminants. Differentially enriched proteins were calculated using empirical Bayes moderated *t-*statistics, using the DEP package^59^ for Bioconductor and R. Normalization of LFQ intensities was used with the variance stabilization transformation (VSN) method^60^ followed by imputation for missing values. *P*-values were corrected for multiple testing using the Benjamini–Hochberg method. For Proteome Discoverer, carbamidomethylation was set as a fixed modification, and oxidation of methionine, N-terminal acetylation, N-terminal methionine loss, and N-terminal methionine loss + acetylation were set as variable modifications. MS1 Orbitrap tolerance was set to 10 ppm and MS2 iontrap tolerance to 0.6 Da. A maximum of two miscleavages per peptide was allowed. The search was made against the UniProtKB/SwissProt *Homo sapiens* proteome (UP000005640, 2022-03-01), including a list of possible contaminants. Quantitative abundances were normalized to the same total peptide amount per sample and scaled so that the average abundance per protein and peptide was 100. Pairwise ratio was used for ratio calculation, and a replicated-based resampling method was used as imputation. A *t*-test based on background proteins was used as the statistical test to find significant changes.

### Structural modeling

The amino acid sequence of human C16orf87 (UniProtKB accession number Q6PH81) or C16orf87 together with HDAC1 (UniProtKB accession number Q13547) and MIER1 (UniProtKB accession number Q8N108) were used as input for structural prediction with AlphaFold3^12^. For each prediction, five structural models were generated, and the model with the highest internal AlphaFold3 confidence ranking was selected for further analysis. To account for the zinc-binding motif, a single zinc ion was included in the C16orf87 monomer modeling, allowing coordination with the cysteine residues near the N-terminus. In the complex modeling, two zinc ions were included to allow both cysteine coordination and the HDAC1 active site. Waggawagga^13^, the MPI Bioinformatics Toolkit^21^, and CoCoNat^22^ were used for analysis of possible coiled-coil structures. All software programs were run with default settings, and where supported, the window size was set to 21 residues. For analysis of the predicted C16orf87–HDAC1–MIER1 complex, PICKLUSTER^34^ and AlphaBridge^36^ were used. AlphaBridge was set to the default (0.7) medium confidence level cut-off. For evaluation of a possible C16orf87 homodimer, AlphaFold-Multimer v3^31^ was run through ColabFold v1.6.0 using MMseqs2 for MSA generation^61^. Template search against pdb100 was enabled. The number of recycles was set to 6. ipSAE was calculated using a Python script^33^, with both the PAE cutoff and distance cutoff set to 15 Å. All protein structure figures and the supplementary video were generated using ChimeraX (version 1.8, https://www.rbvi.ucsf.edu/chimerax^62^) or PyMOL (PyMOL Molecular Graphics System, version 2.3.0, https://www.pymol.org).

### Scratch assay

An equal number of the Panc-01^WT^ and Panc-01^KO^ cells were seeded on a 6-well plate and were cultured to confluency. Perpendicular scratches were made using 200 µL filter tips, and the growth media was replaced every 12 h. Images were taken for each well at 0, 6, 12, and 24 h using a Nikon D300. The area of the wound was measured for all the fields of each well using ImageJ. Statistical significance was set at *P* ≤ 0.05, and the experiment was done in biological triplicate.

### Cell viability assay

Panc-01^WT^ and Panc-01^KO^ were grown for 48 h, and a cell viability assay was performed with a flow cytometer (Cytoflex S, Beckman Coulter) using Annexin-V-FITC and DRAQ7 as the detection dyes. Data from 10,000 events per sample were collected and analyzed using the FlowJo software (Ashland, OR). A *t-*test was used for determining significance (*P* ≤ 0.05, n = 3).

### Cell proliferation assay

The Click-iT Plus EdU flow cytometry kit (Thermo Fisher Scientific) was used to assess differences in cell proliferation. Briefly, the cells were incubated for 2 h with EdU-A647, a nucleoside analog containing an alkyne, to incorporate EdU-A647 into the cells. Cells were trypsinized, washed with PBS containing 1% BSA, and then the cell pellet was treated with the Click-iT fixative. The cells were incubated for 15 min at room temperature in the dark. The cells were permeabilized and washed with Click-iT saponin-based buffer. The Click-iT EdU A647 reaction cocktail containing PBS, coper protectant, fluorescent dye picolyl azide, and EdU A647 was added to the cells, following incubation for 30 min in the dark at room temperature. A saponin-based permeabilization and wash buffer was added to the cells before reading the Alexa Fluor 647 picolyl azide at 633/635 nm excitation with a red emission filter (660/20 nm). The statistical significance was determined in a manner similar to the cell viability assay.

### RNA isolation and qRT-PCR

RNA was isolated using TRIreagent (Merck) and contaminating genomic DNA was removed with the RapidOut DNA removal kit (Thermo Fisher Scientific). About 1 µg of total RNA was converted to cDNA using random primers and Maxima H minus reverse transcriptase (both from Thermo Fisher Scientific). qRT-PCR reactions were carried out with HOT FIREPOL EvaGreen qPCR Supermix (Solis BioDyne) using a QuantStudio 6 Flex Real-Time PCR System (Applied Biosystems). Every sample was run in triplicate and normalized to the expression of the housekeeping gene *18S rRNA or HRPT1*^52,63^. Validated qRT-PCR primers (*WWOX* (HP212154), *NCOA7* (HP233599), *HINT3* (HP217026), and *C16orf87* (HP200968)) were purchased from Origene. Results are expressed as the mean ± standard deviation (SD). An unpaired* t*-test was used to test for statistical significance (****, *P* ≤ 0.0001; ***, *P* ≤ 0.001; **, *P* ≤ 0.01; *, *P* ≤ 0.05).

### ATAC-seq

Approximately 50,000 viable Panc-01^WT^ or Panc-01^KO^ cells were disrupted on a lysis buffer (10 mM Tris pH 7.4, 10 mM NaCl, 3 mM MgCl_2_, 0.1% NP40, 0.1% Tween-20, and 0.01% Digitonin). The cell nuclei were collected and rinsed in a washing buffer (10 mM Tris pH 7.4, NaCl 10 mM, 3 mM MgCl_2_, 0.1% Tween-20). The tagmentation reaction was carried out by the TDE1 enzyme in a buffer containing PBS, 1% digitonin, and 10% Tween-20. The MinElute purification kit (Qiagen) was used to obtain purified, concentrated DNA.

Library preparation was performed with the Omni-ATAC method^64,65^. Once the final library was obtained, a purification step using AMPure XP beads was performed before concentration and assessment of fragment size. A quality-control step consisting of size distribution and yield estimation is performed before library sequencing. The libraries were pooled and sequenced on a NextSeq 2000 sequencer (Illumina) using one P2-100 flowcell (2 × 50 bp set-up), resulting in approximately 60–100 million read pairs at the individual sample level across the six samples. The sequencing results from the ATAC-seq analysis performed by the NGI platform were analysed by the Nextflow pipeline NF-core/atacseq version 2.1.2 (https://nf-co.re/atacseq)^66^. Briefly, sequence adapters were trimmed using Trim Galore! and aligned to the human genome (GRCh38) using the BWA package. The Picard package marked duplicates and merged alignments from multiple libraries into a single sample. Mitochondrial DNA, duplicates, unmapped regions, and other unwanted sequences were filtered using SAMtools, Pysam, and BAMTools. Normalized bigWig files were generated by BEDTools, and bedGraphToBigWig. Genome-wide enrichment was performed using deepTools, and peaks were identified using MACS2. Differential accessibility analysis was performed by DESeq2, and Homer was used to annotate gene features. The statistical analysis was performed using a negative binomial generalized linear model and tested and adjusted by the Wald test and the Benjamini and Hochberg method, respectively. A difference between peaks in Panc-01^WT^ and Panc-01^KO^ was considered significant when the *p*-adjusted value ≤ 0.05. Data were visualized using the Integrative Genome Viewer (IGV), and additional tracks for HDAC1, HDAC2, MIER1, MIER2, MIER3, and H3K27ac markers were obtained from ChIP-Atlas.

### Zebrafish growth conditions

Adult AB zebrafish (*Danio rerio*) were kept at a maximum density of 5 fish/L in 5–10 tanks at a controlled water temperature of 28.5 °C and a light/darkness cycle of 10/14 h with a 30 min dimming period in between. Adults were fed with > 400 μm micro-extruded food pellets (ZEBRAFEED, Sparos) in the morning and were supplemented with live rotifers in the afternoon. Adults were purposely bred to generate larvae for the following experiment. All zebrafish adult caretaking procedures described here were approved by the Uppsala Board for Animal Research (Djurförsöksetiska nämnd) of the Swedish Ministry of Agriculture (Jordbruksverket) under permit number Dnr 5.8.18-13680/2020. The larvae used in the experiment were less than 5 days old. Methods to minimize pain and suffering were used for all experiments. The ethical treatment and welfare of the zebrafish larvae were ensured, as well as the scientific integrity of the research. This study was performed in accordance with relevant guidelines and regulations. All methods are reported in accordance with ARRIVE guidelines. Larvae at this developmental stage are sexually undifferentiated; therefore, sex was not reported.

### Zebrafish genome editing and developmental monitoring

We first identified the zebrafish gene *C7H16orf87* as an orthologue of the human C16orf87, with 76% sequence identity (Fig. 1a) between the human and zebrafish amino acid sequences. CRISPOR (https://crispor.gi.ucsc.edu/) was used to design two gRNA sequences to target exon 2 (to halt transcription, 5′-GAAGACGTAGCCACAAGGA-3′) and exon 3 (to target a functional domain as done in human cells, 5′-ATTCAGATCCAATCCGCAGA-3′) with low off-target scores. About 50 μM of 2-part gRNA (tracrRNA + crRNA) was combined with Alt-R S.p Cas9 nuclease (IDT Technologies). Phenol red (Waterline Technologies Inc., to visualize microinjections) and water were added to reach a 5 μM gRNA:Cas9 RNP complex. Eggs were collected from adult AB zebrafish in breeding tanks and randomly distributed into two groups to be microinjected within 30–60 min of fertilization. Since microinjections, DNA damage, and DNA repair mechanisms all affect early embryonic development, we microinjected a gRNA targeting the *kita* gene, which affects melanocyte migration and pigmentation^67^, in both groups. Eggs were kept in petri dishes containing methylene blue water from day 0 until day 3, at a density of 60 eggs per dish, at 28.5 °C, with the same light/darkness cycle as adults. On day 3, successfully targeted larvae were identified using a stereoscope based on lower pigmentation rates. Using stereoscopy, we obtained mutation rates of at least 90.6% at exon 2 and 93.8% at exon 3 based on a subset of 32 targeted larvae and 8 sibling controls collected from the same injection round. The efficiency in mutation rate was determined as described in^68^. No larvae within this subset were misclassified, and only 3 and 2 samples failed to amplify from each target. Before imaging, the successfully micro-injected, 3-day-old larvae were anesthetized with MS-222 (0.125 μg/mL, Sigma) and distributed in a flat-bottomed 96-well plate. After imaging, larvae were dispensed into plates containing 0.267 µg/µL of cold MS-222 solution for euthanasia. A vertebrate automated screening technology (VAST) bioimager system (Union Biometrica Inc.) was used to aspirate individual larvae from each well and place them inside a 600 ± 25 μm borosilicate capillary with lateral orientation in the field of view of a brightfield camera with 10 × magnification. For each larva, we acquired twelve brightfield images at each 30° of rotation, including two dorsal/ventral and two lateral views. Imaging was performed automatically, without user input and in a sequence alternating between crispants and controls. Images were analyzed using a deep learning-based neural network as described elsewhere^68^. This neural network segments larvae from the background and quantifies their body length, dorsal and lateral area, and eye area. It also draws a line through the center of the eye and ear, and across the back contour. This allows us to calculate the angle between the head and the back, which on day 3 is a proxy for the developmental stage^41^.

Quality control of the data was performed by removing, for each outcome, outliers with values outside the mean ± 5 × SD window, and larvae with discrepancies between left/right top/bottom images, since they typically reflect incorrect rotation of the capillary in which the larvae are imaged. All traits were inverse normally transformed to a mean of 0 and SD of 1 before the statistical analysis. Linear regression was performed to determine the effect of mutations in *C7H16orf87* on the traits of interest, adjusted for the time of imaging. Effects on the dorsal and lateral body area were additionally adjusted for body length. Stata MP v16 was used for data management and the statistical analysis of zebrafish data.

## Supplementary Information

Supplementary Information 1.

Supplementary Video 1.

Supplementary Information 2.

Supplementary Information 3.

Supplementary Information 4.

## Data Availability

The MS proteomics data have been deposited to the ProteomeXchange Consortium (https://proteomecentral.proteomexchange.org) via the PRIDE partner repository^69^ with the dataset identifiers PXD069175 (siRNA C2C12), PXD069216 (siRNA Panc-01), PXD069192 (siRNA MiaPaCa-2), PXD069174 (IP-MS HeLa), PXD069221 (Panc-01 KO /Panc-01 WT), and PXD069222 (Phosphoproteomics C2C12). The ATAC-seq data have been deposited in the NCBI’s Gene Expression Omnibus^70^ and are accessible through GEO Series accession number GSE308337 (https://www.ncbi.nlm.nih.gov/geo/query/acc.cgi?acc=GSE308337).
