# Supplementary material for: The C16orf87 protein is a subunit of the MIER corepressor complex controlling embryonic development and cell migration
Source: Sci Rep. 2026 Apr 30;16:13907. doi: 10.1038/s41598-026-50740-7 (PMC13133221; doi:10.1038/s41598-026-50740-7)
Supplement: Supplementary file 1 — Supplementary Information 1. [file 41598_2026_50740_MOESM1_ESM.pdf]

Supplementary materials and methods

Validation of the CRISPR-Cas9 genome editing

Genomic DNA from Panc-01^{KO} cells treated with the crRNA and SpCas9 protein RNP complex was isolated using the DNeasy Blood & Tissue Kit (Qiagen). Semi-quantitative PCR was performed on the isolated genomic DNA using forward primer (5’-GGCGGGCTGTGGGTCGCGAG-3’) and reverse primer (5’-GTGGGGCTCCAGACAGCC-3’) in the presence of Pfu DNA polymerase (Thermo Fisher Scientific).

MS-based phosphoproteomics and data analysis

TMT Labeling and Peptide Fractionation

A phosphoproteomics analysis was carried out for the LIF-treated and untreated C2C12 cells (Supplementary Fig. 3b). A total of 300 µg of peptides per sample were labeled using the TMT-10plex reagent (Thermo Fisher Scientific) in 50 mM HEPES (pH 8.5). Labeling efficiency was verified via LC-MS/MS analysis of pooled QC aliquots. After verification, labeling reactions were quenched with 5% hydroxylamine. Combined labeled samples were desalted using C18 cartridges and dried. Peptide fractionation was performed in two stages to generate fractions that were recombined to increase peptide resolution, as previously described (1). Briefly, we first used the OFFGEL Fractionator kit to separate peptides on an OFFGEL system G3100 (Agilent) and using a High-pH reversed-phase chromatography kit (Thermo Fisher Scientific) to generate fractions with recombined peptides with different isoelectric points to increase peptide resolution as previously described (1).

Phosphopeptide enrichment

Phosphopeptides were enriched using the Fe-NTA Phosphopeptide Enrichment Kit (Thermo Fisher Scientific). Ni-NTA agarose beads were stripped with 100 mM EDTA, recharged with 10 mM $FeCl_3$, and equilibrated in a 1:1:1:1 slurry with methanol (MeOH), acetonitrile (MeCN), 0.01% acetic acid, and beads. Peptides were reconstituted in 80% MeCN/0.1% trifluoroacetic acid (TFA) and incubated with the beads. Lastly, phosphopeptides were desalted on C18 stage tips and eluted with

potassium phosphate buffer, then dried and reconstituted in 0.1% formic acid before LC-MS/MS.

LC-MS/MS and Data Analysis of TMT-labelled Phosphopeptides

Peptides were fractionated using an EASY-nLC 1000 (Thermo Fisher Scientific) coupled to an Orbitrap Fusion Tribrid mass spectrometer (Thermo Fisher Scientific). Peptides were resolved on a 75 μm x 50 cm, 2 μm particle size, EASY-Spray PepMap RSLC C18 column, and analyzed under data-dependent acquisition mode. TMT-labeled peptides were fragmented using higher-energy collisional dissociation (HCD), with MS2 resolution set to 50,000 at m/z 200 to resolve reporter ions. Raw data were processed with Proteome Discoverer version 2.5 (Thermo Fisher Scientific). Canonical sequences of the human proteome (UNIPROT UP000005640) were used as the proteome database. TMT labelling efficiency was determined by database searches allowing for variable modifications on N-termini and lysines. Protein-level quantification was based on median reporter ion intensities from filtered high-purity precursor PSMs, corrected for isotopic impurities. Phosphoproteomics analysis was performed using the PhosphoRS node to determine phosphorylation location and probability (2). Phosphopeptide ratios of LIF-treated versus control samples ≥1.5 and phosphoRS probability ≥95% were considered for further analysis if their respective protein ratio was equal to 1.

His-tagged protein purification

The 6xHis-MS2- (4) and 8xHis-C16orf87-expressing plasmids were transformed into BL21(DE3) competent cells, bacterial cultures were grown to OD_{600}=0.5, and protein expression was induced with 1 mM IPTG at 37 °C for 3 h. Soluble proteins were purified as previously (4) and dialyzed against buffer H (20 mM Tris-HCl pH 8.0, 100 mM NaCl, 10% glycerol, 1 mM $MgCl_2$). Dialyzed soluble proteins were stored at -80°C.

HDAC1 assay

HDAC1 Fluorogenic Assay (BPS Biosciences) was used to monitor HDAC1 activity according to the manufacturer's protocol. Shortly, increasing amounts of the purified His-MS2 and His-C16orf87 (8, 80, and 800ng) proteins and the recombinant HDAC1

(10 ng) were used in the reactions. Reactions were incubated at 37°C for 30 min, and the produced fluorophore was detected with a TECAN M200 plate reader (Tecan).

References

1. Mertins, P., Tang, L. C., Krug, K., Clark, D. J., Gritsenko, M. A., Chen, L. *et al.* (2018) Reproducible workflow for multiplexed deep-scale proteome and phosphoproteome analysis of tumor tissues by liquid chromatography-mass spectrometry Nat Protoc **13**, 1632-1661 10.1038/s41596-018-0006-9
2. Taus, T., Kocher, T., Pichler, P., Paschke, C., Schmidt, A., Henrich, C. *et al.* (2011) Universal and confident phosphorylation site localization using phosphoRS J Proteome Res **10**, 5354-5362 10.1021/pr200611n
3. Wang, H., Chumnarnsilpa, S., Loonchanta, A., Li, Q., Kuan, Y. M., Robine, S. *et al.* (2009) Helix Straightening as an Activation Mechanism in the Gelsolin Superfamily of Actin Regulatory Proteins Journal of Biological Chemistry **284**, 21265-21269 10.1074/jbc.M109.019760
4. Dauksaite, V., and Akusjärvi, G. (2002) Human splicing factor ASF/SF2 encodes for a repressor domain required for its inhibitory activity on pre-mRNA splicing Journal of Biological Chemistry **277**, 12579-12586 10.1074/jbc.M107867200

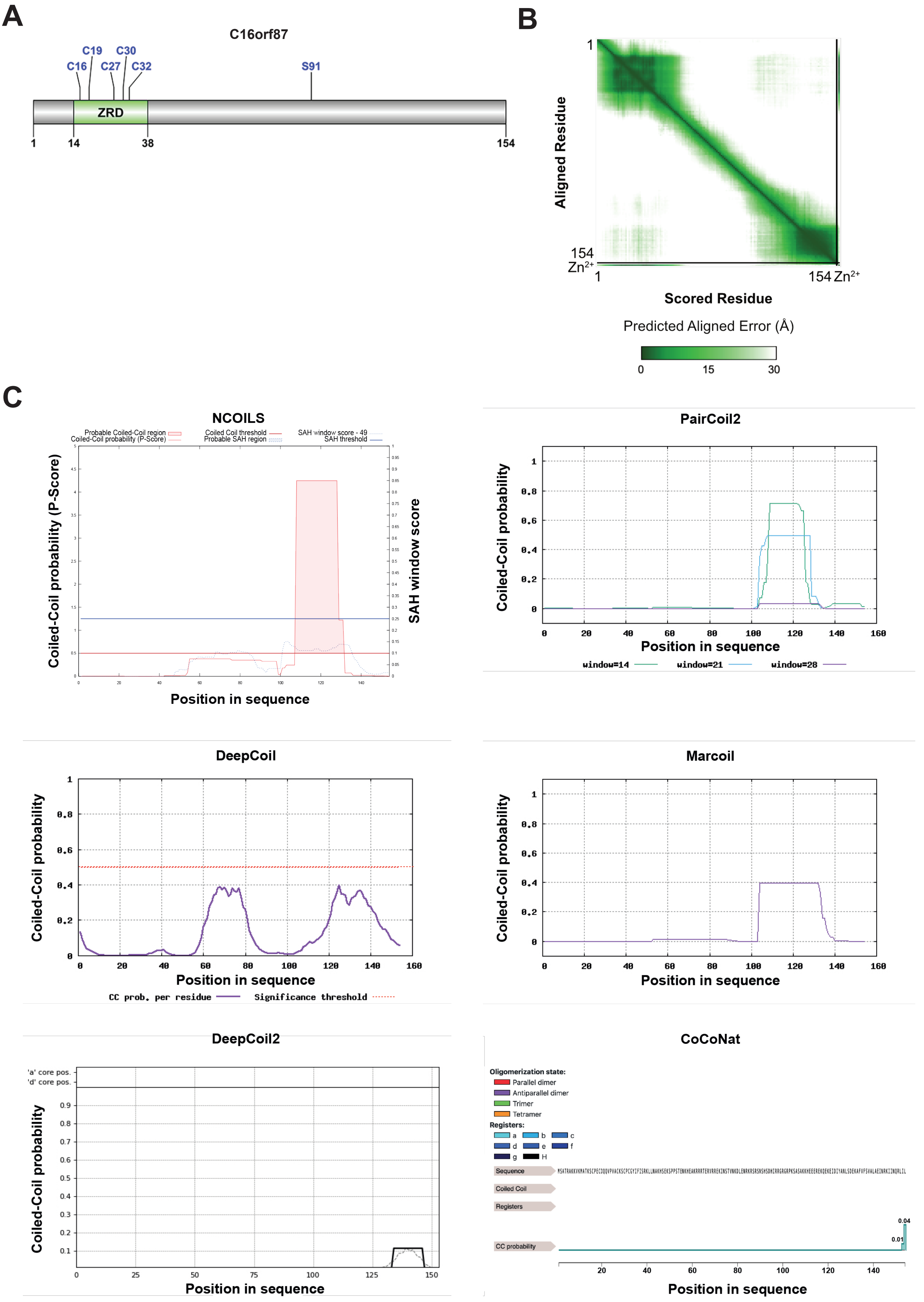

Supplementary Fig. 1

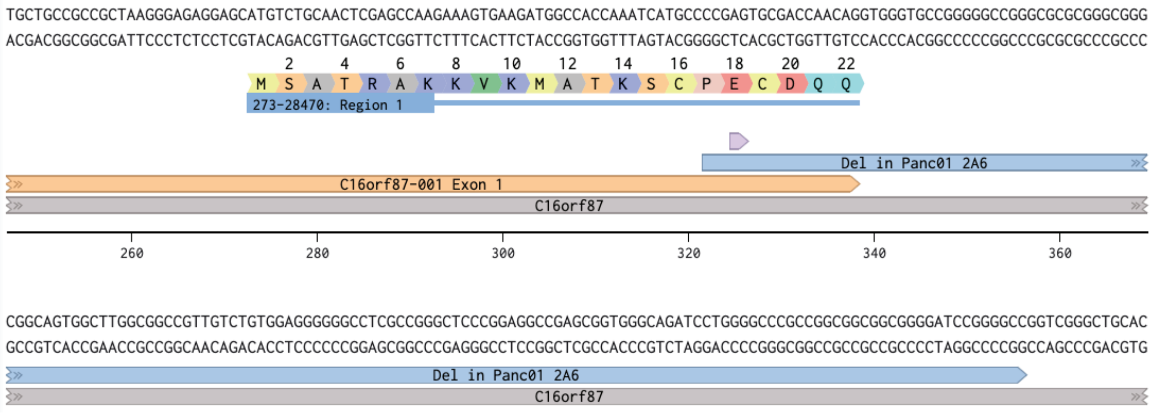

Supplementary Fig. 2

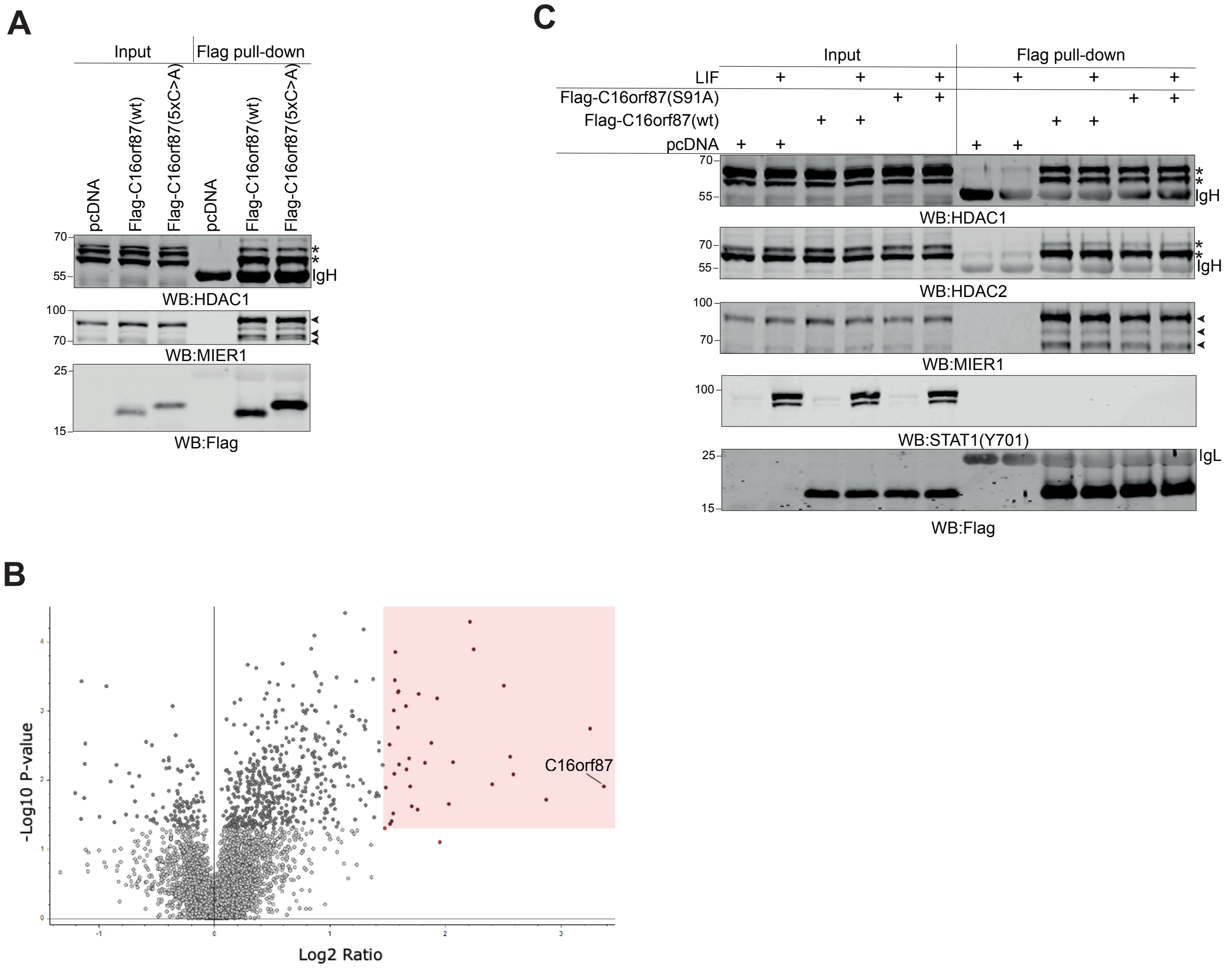

Supplementary Fig. 3

A

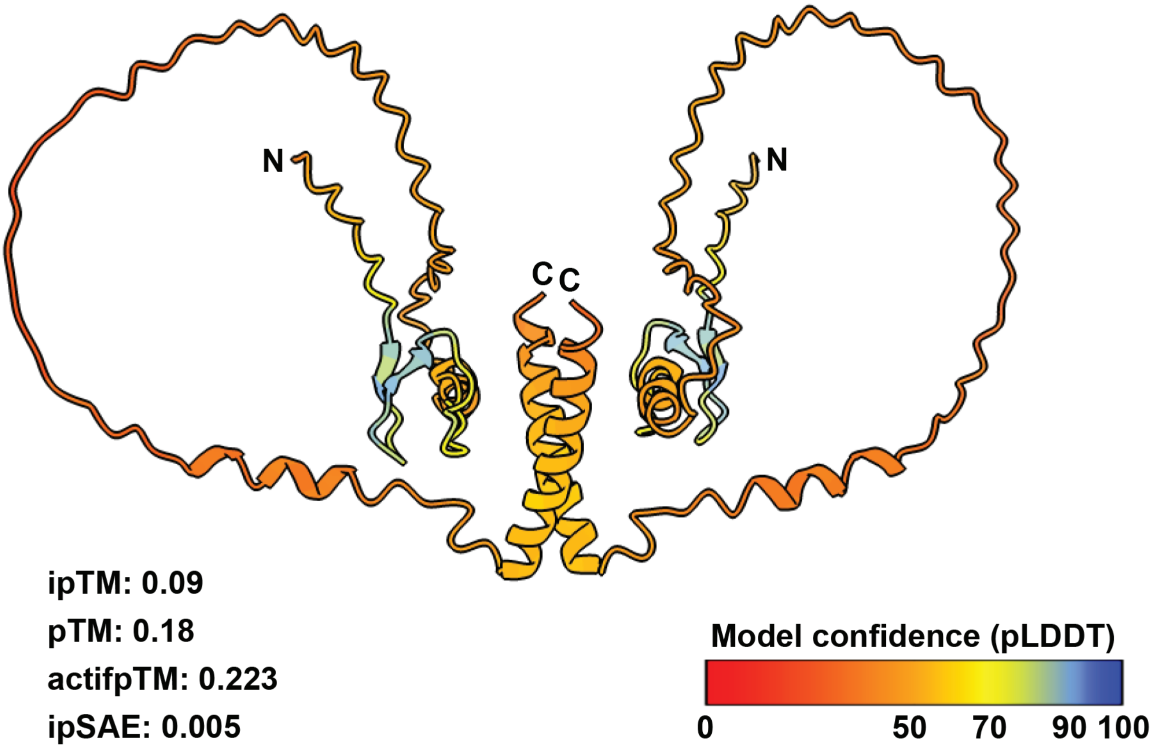

B

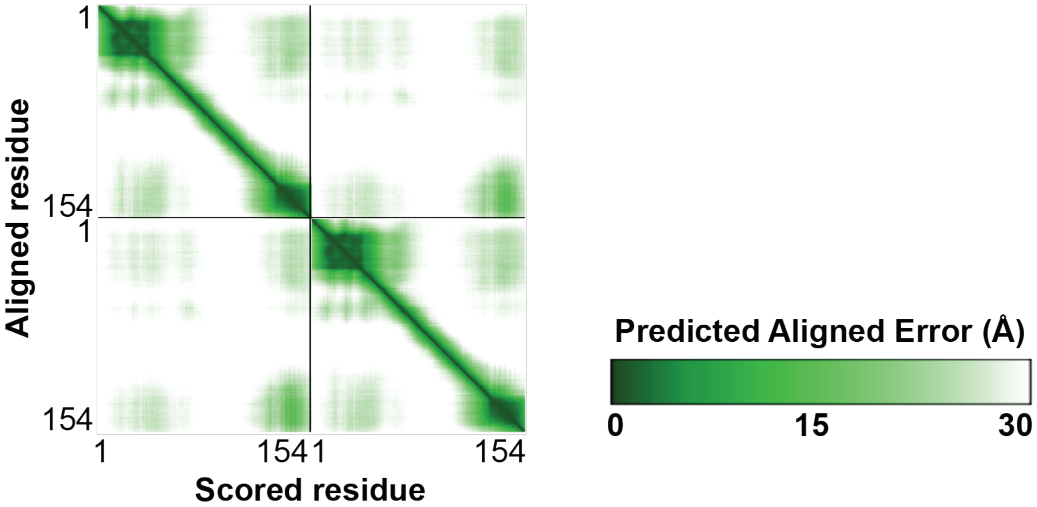

Supplementary Fig. 4

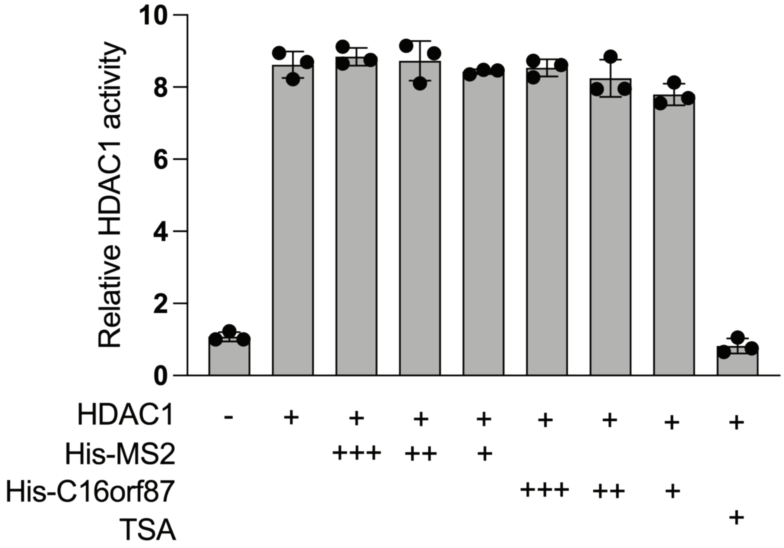

Supplementary Fig. 5

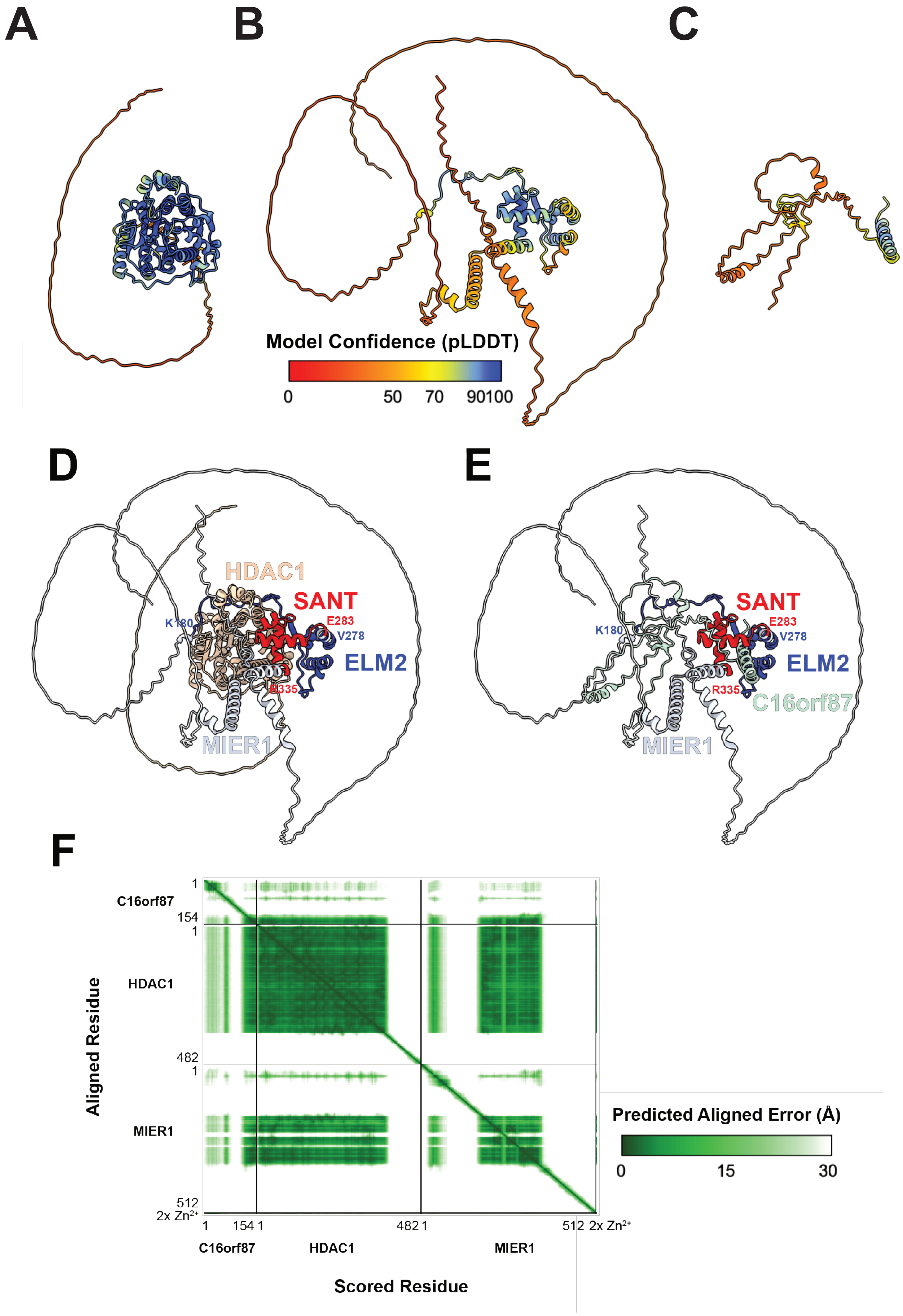

Supplementary Fig. 6

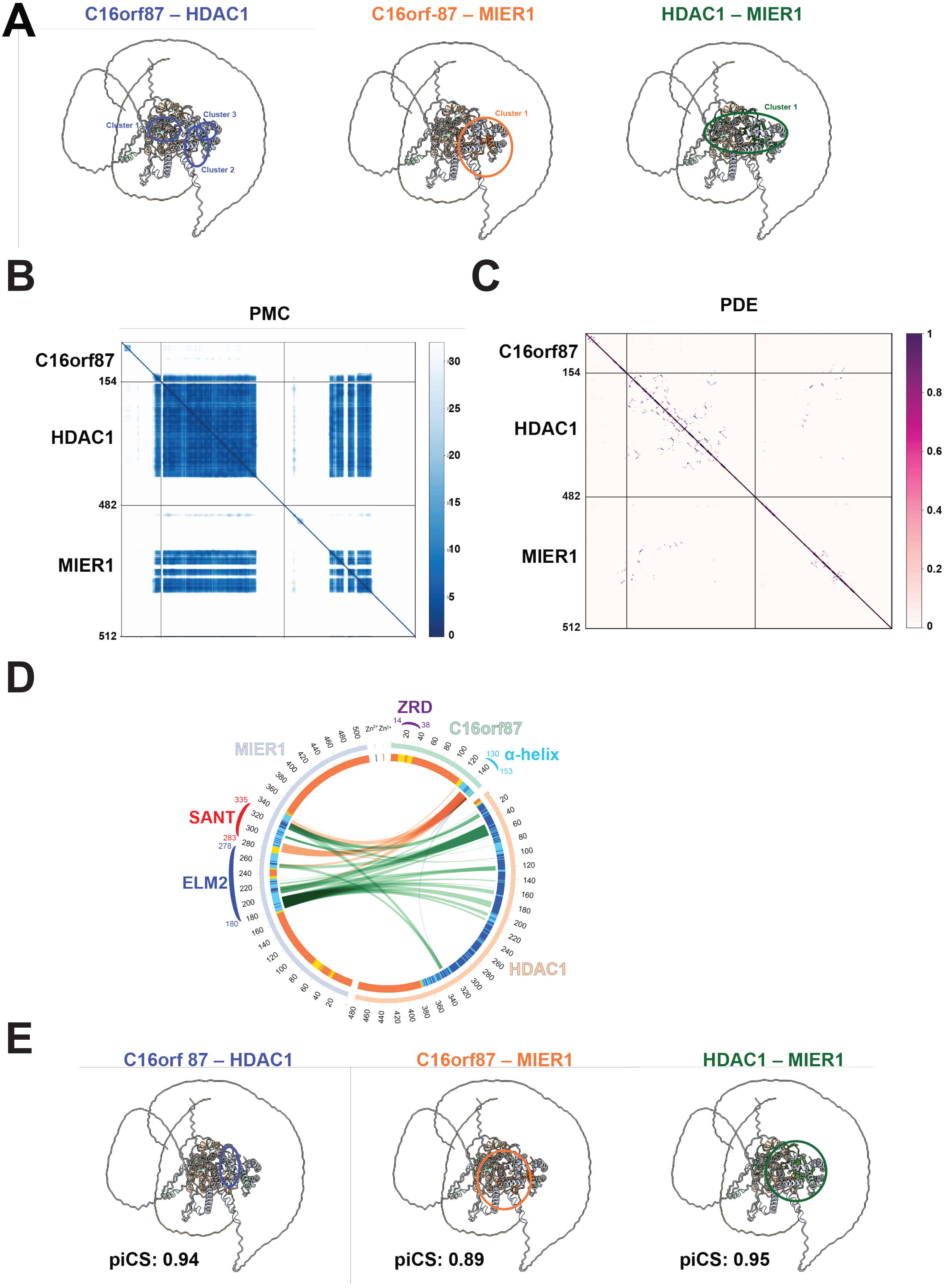

Supplementary Fig. 7

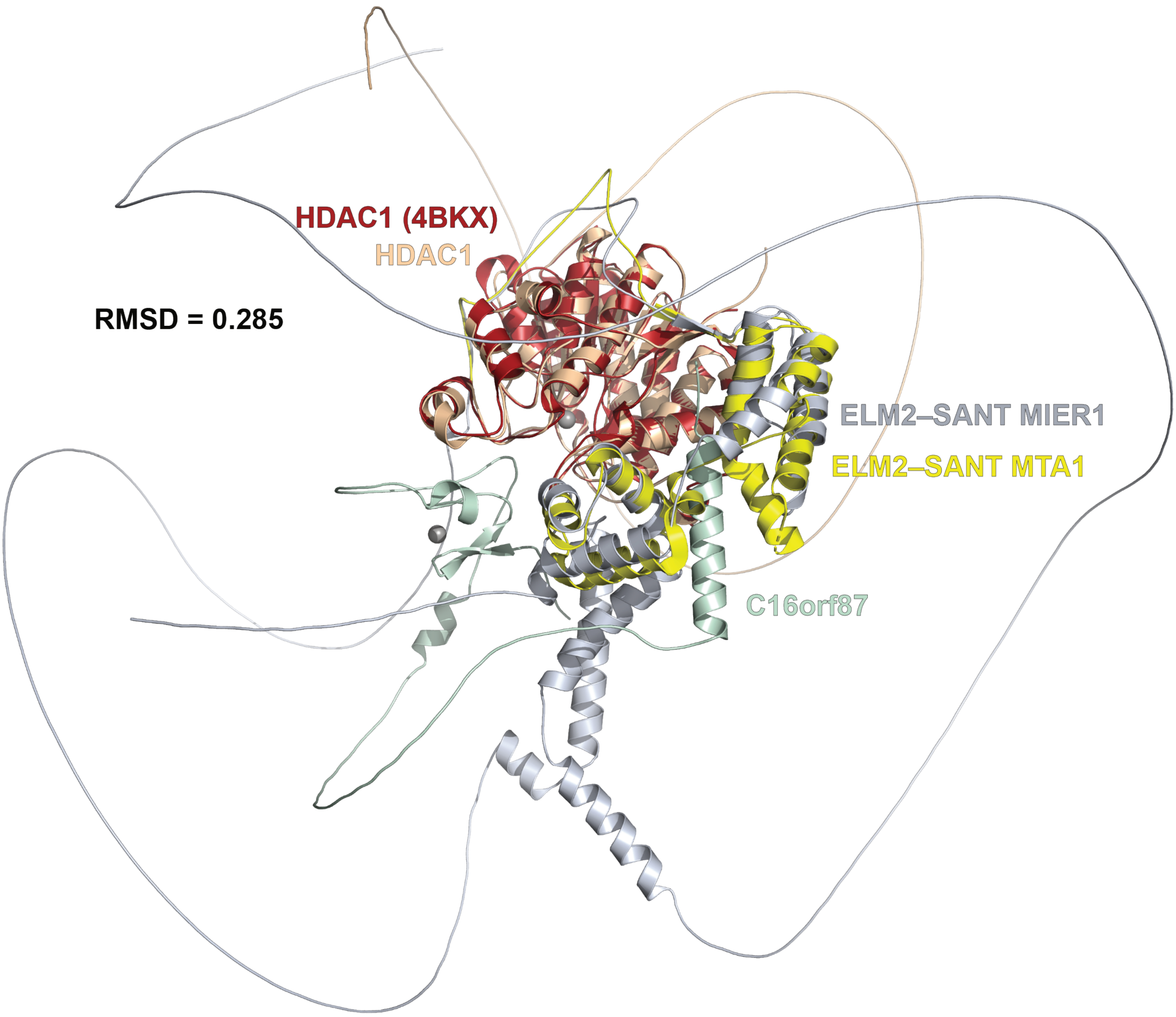

Supplementary Fig. 8

A

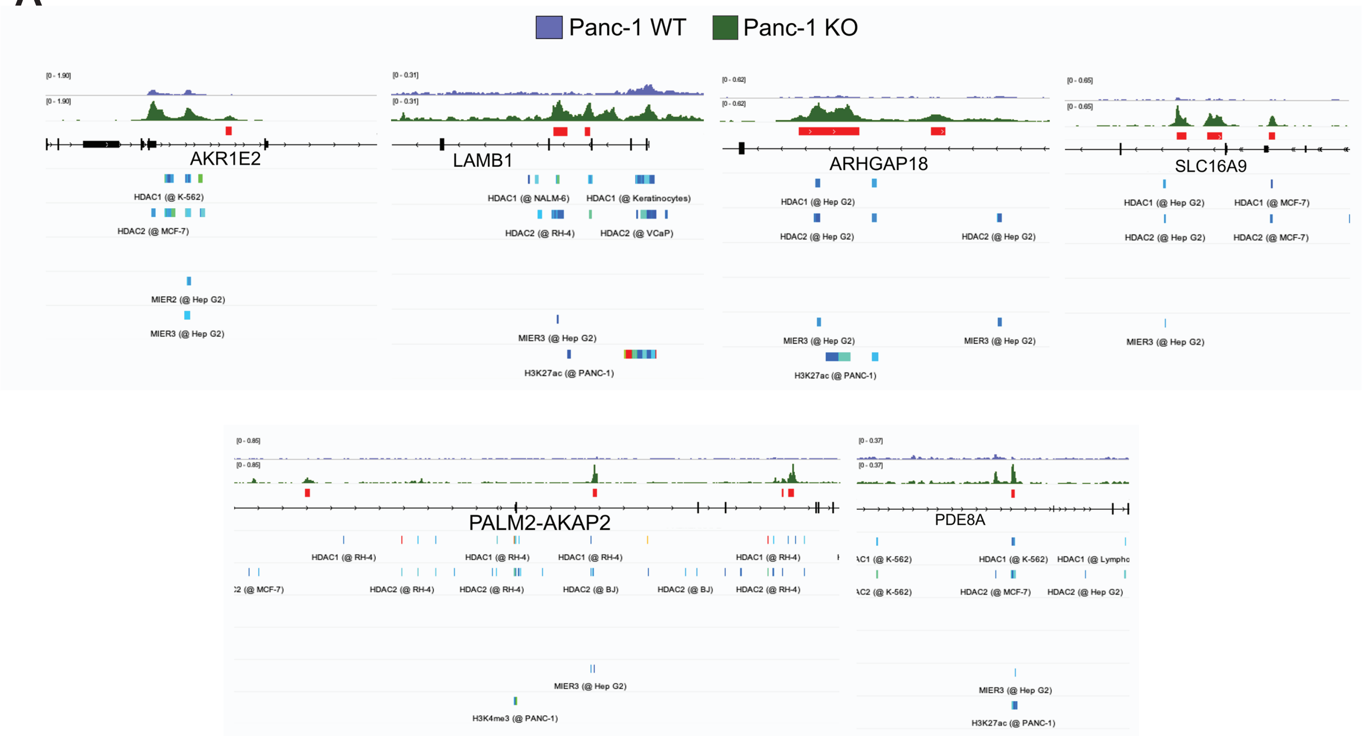

B

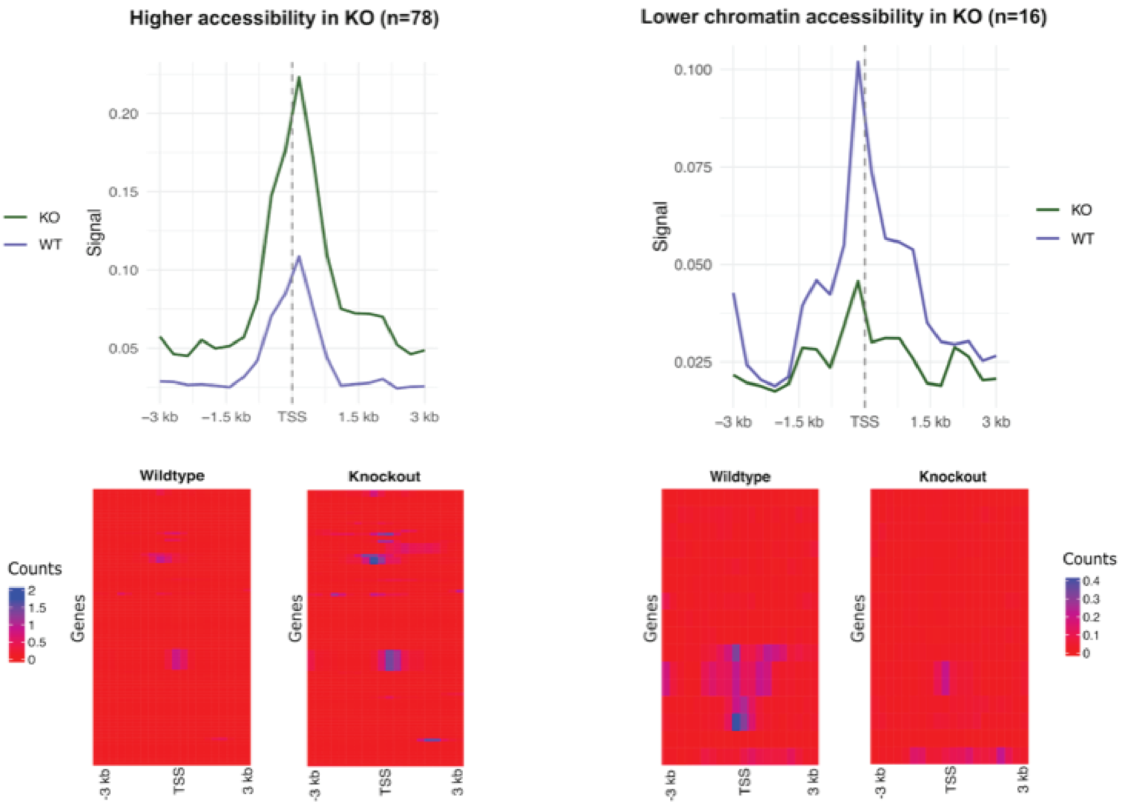

Supplementary Fig. 9

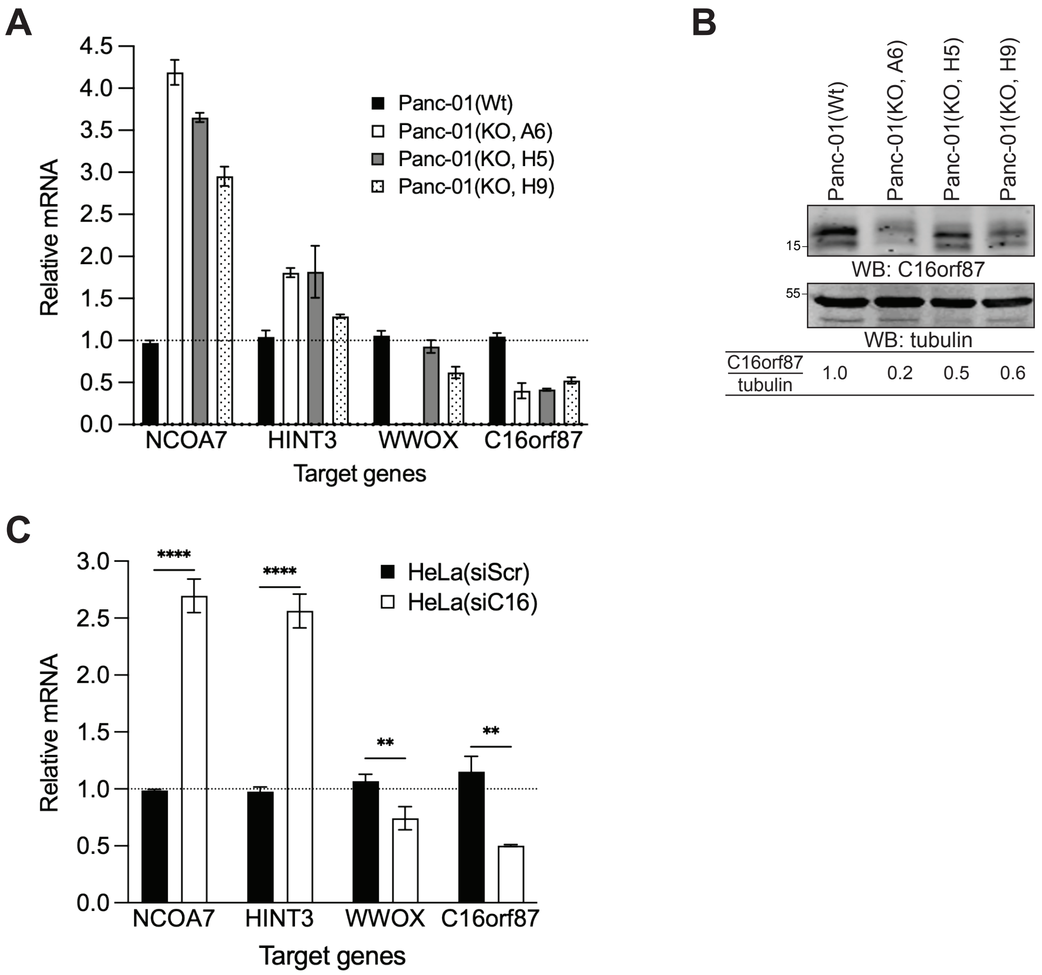

Supplementary Fig.10

Blot shown in the figure:

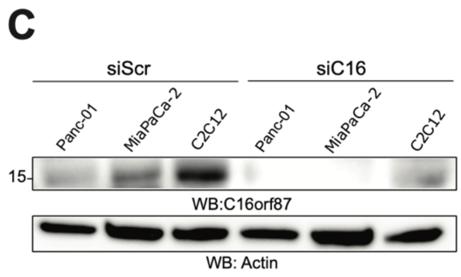

Original picture of the blot:

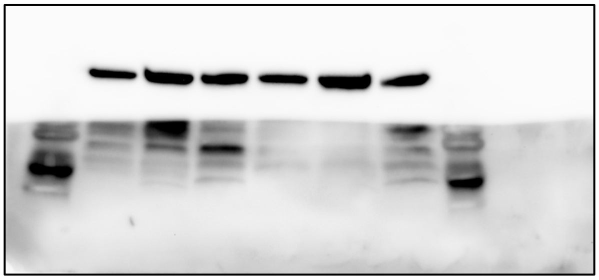

Original blot with labels:

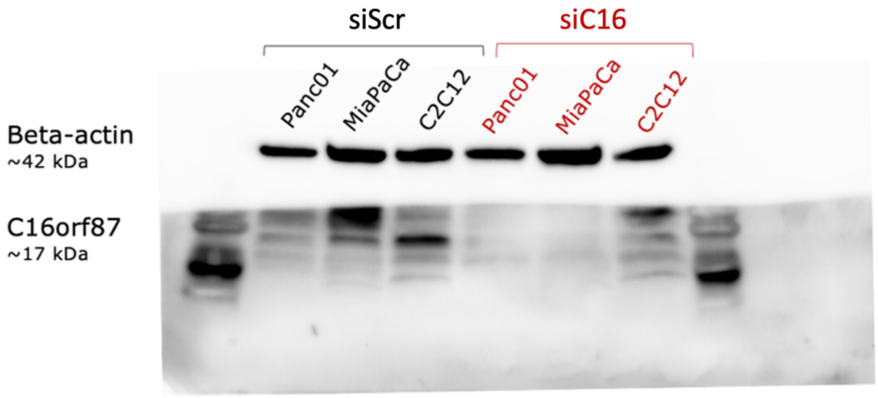

Supplementary Fig. 11

Blot shown in the figure:

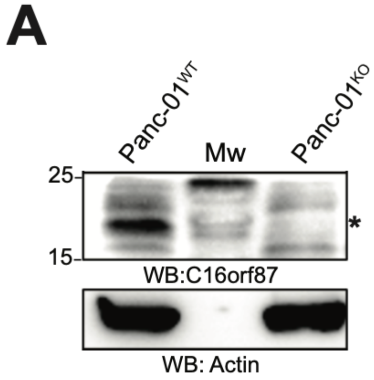

Original blot image:

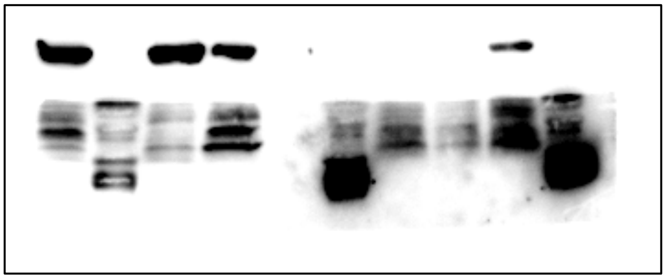

Original blot image with labels

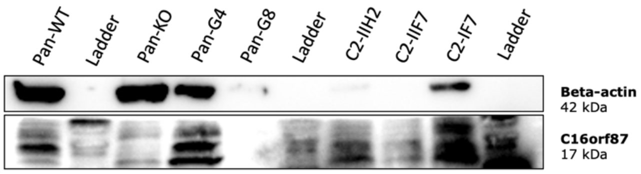

Supplementary Fig. 12

Blot shown in the figure:

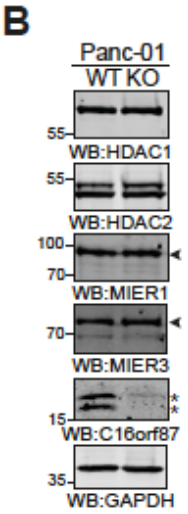

Original blot image:

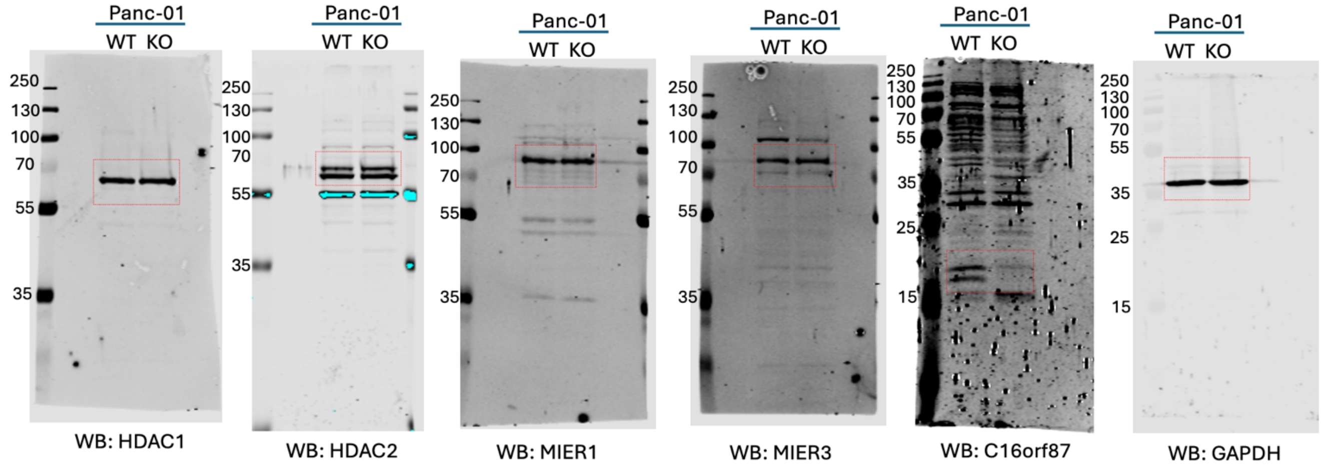

Supplementary Fig. 13

Blot shown in the figure:

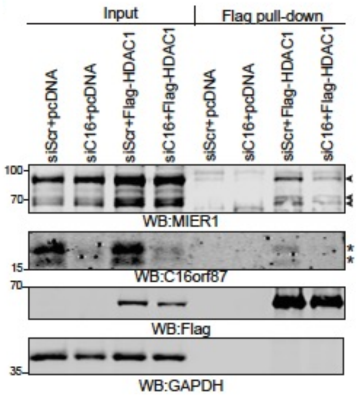

Original blot images

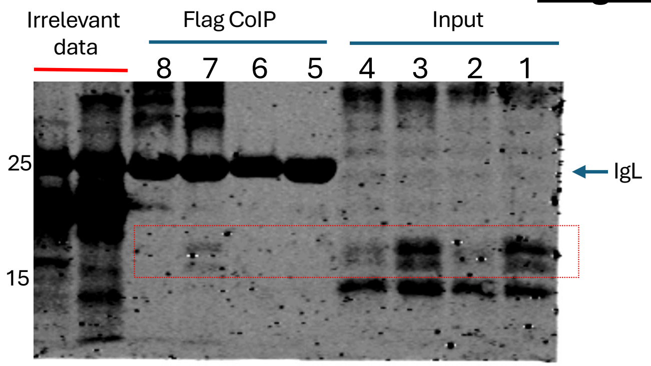

WB: C16orf87 (long expo)

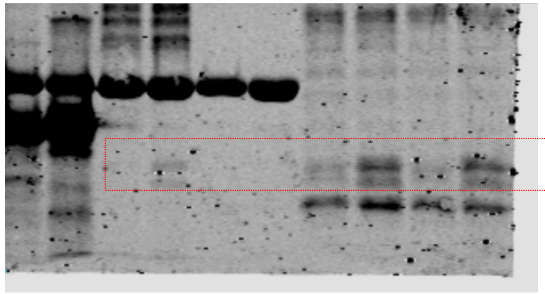

WB: C16orf87 (middle expo)

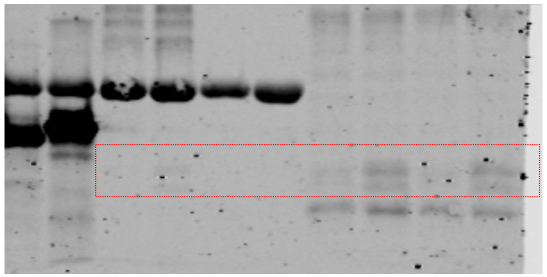

WB: C16orf87 (short expo)

NB! To achieve higher resolution, only part of the original membrane was scanned.

Sample loading on SDS-PAGE

- #1 siScr+pcDNA input
- #2 siC16+pcDNA input
- #3 siScr+Flag-HDAC1 input
- #4 siC16+Flag-HDAC1 input
- #5 siScr+pcDNA Flag CoIP
- #6 siC16+pcDNA Flag CoIP
- #7 siScr+Flag-HDAC1 Flag CoIP
- #8 siC16+Flag-HDAC1 Flag CoIP

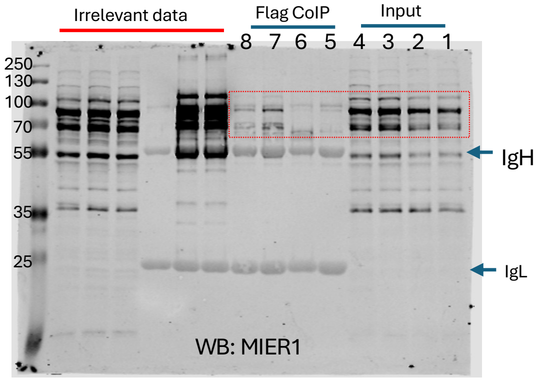

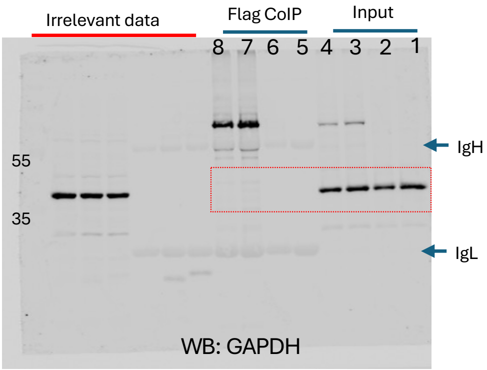

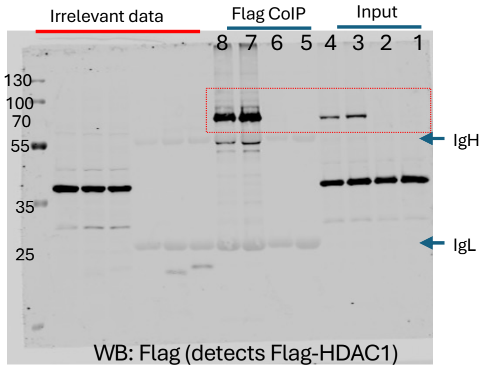

Supplementary Fig. 14

Original scan for Figure 4D

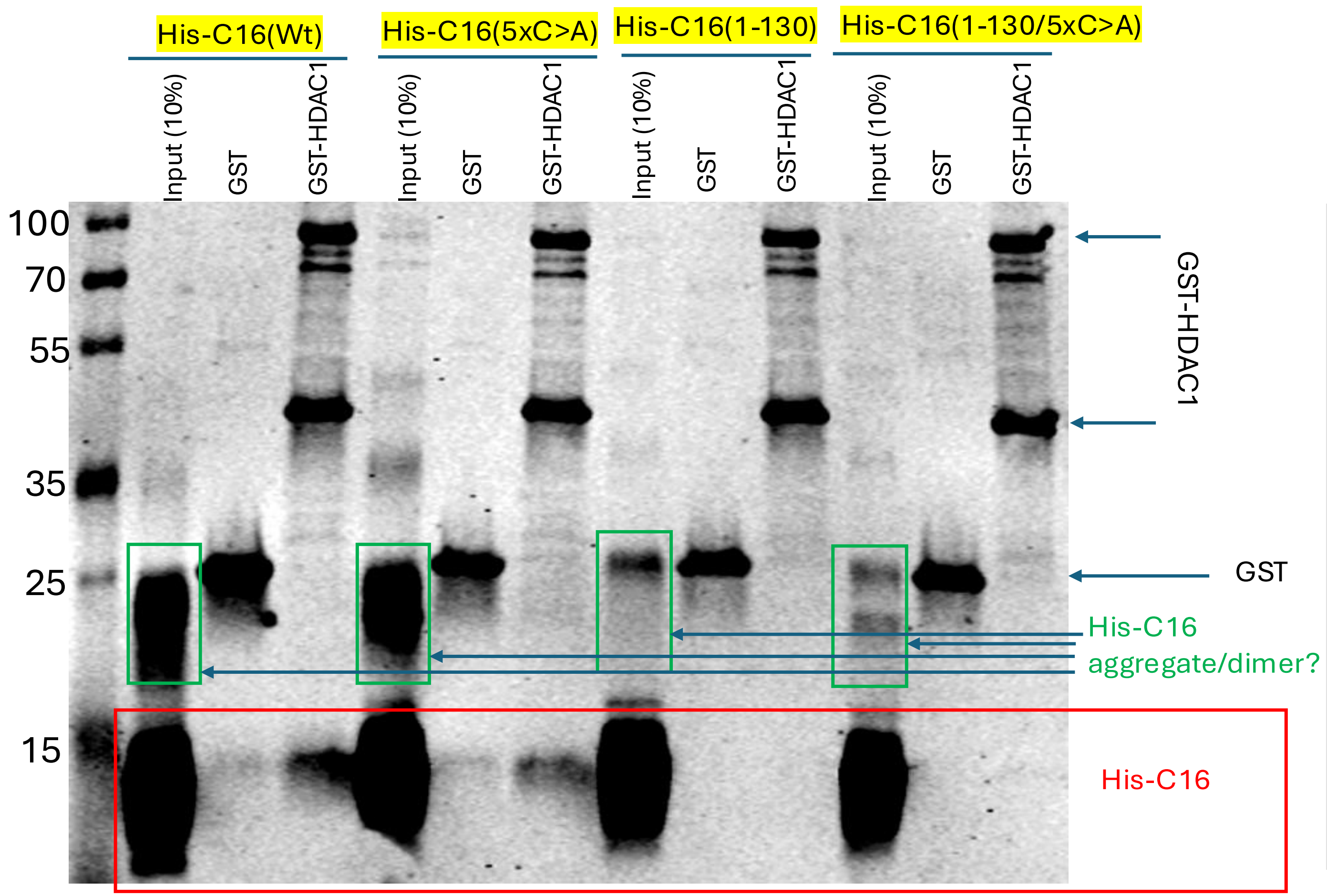

WB: a-His(mouse)/ a-GST(goat) (High expo)

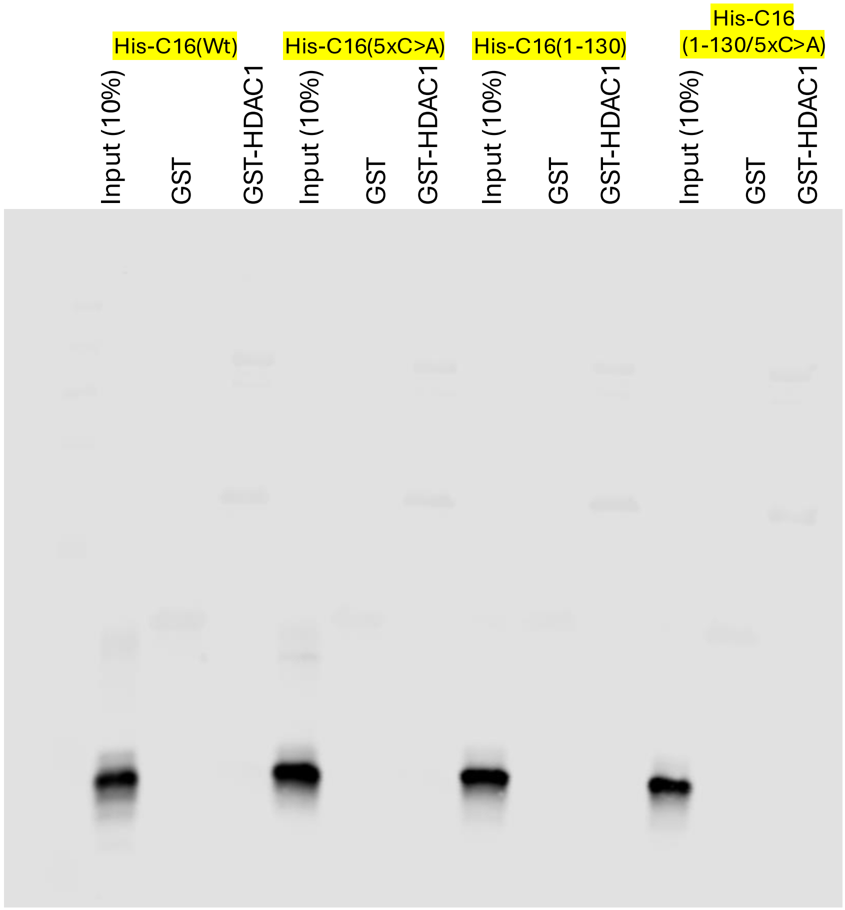

Low expo with a-His antibody

Supplementary Fig. 15

Original scans Supplementary Fig. 10b

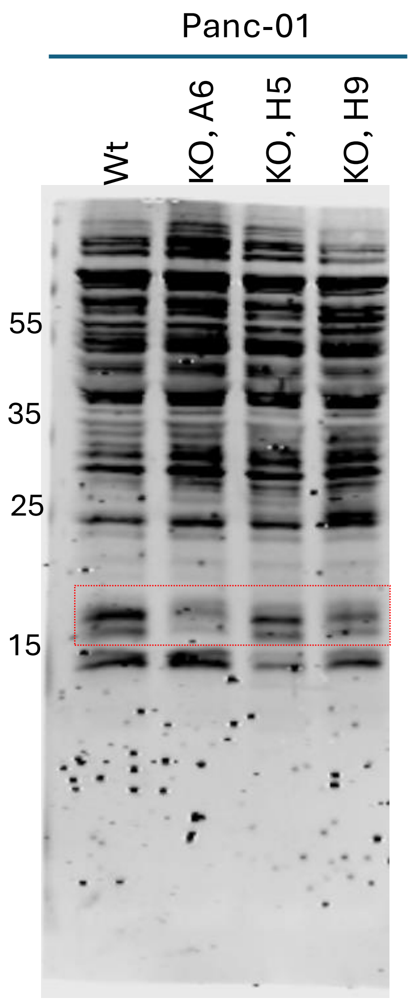

WB: C16orf87

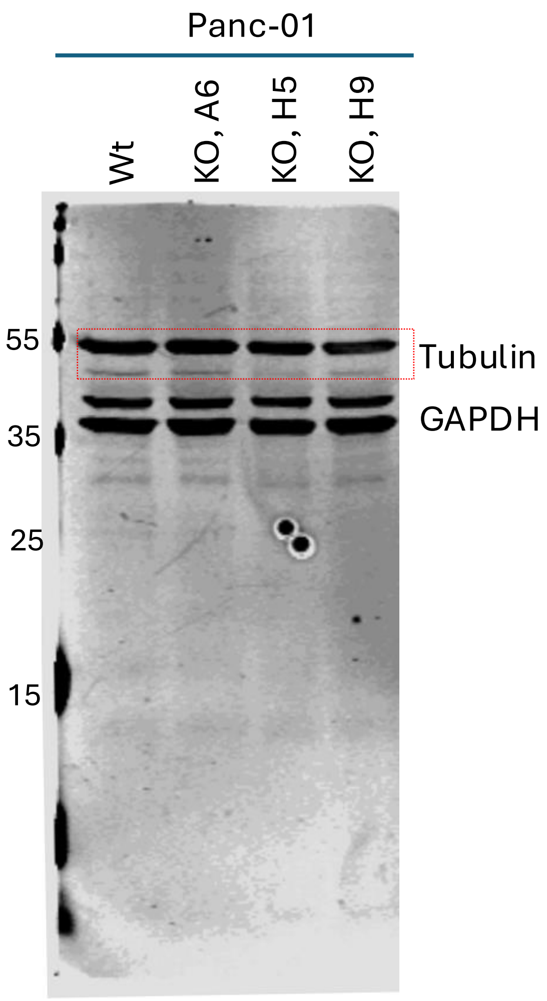

WB: GAPDH & tubulin

Processed scans
Supplementary Fig. 10b

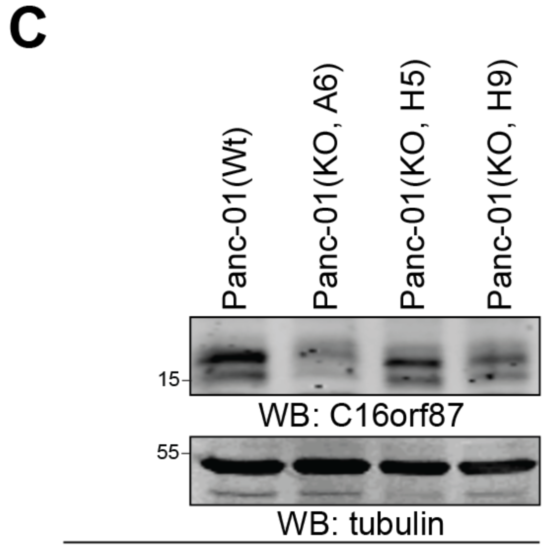

C16orf87 / tubulin	1.0	0.2	0.5	0.6

Supplementary Fig. 16

Original scans for Supplementary Fig. 3A and Supplementary Fig. 3C

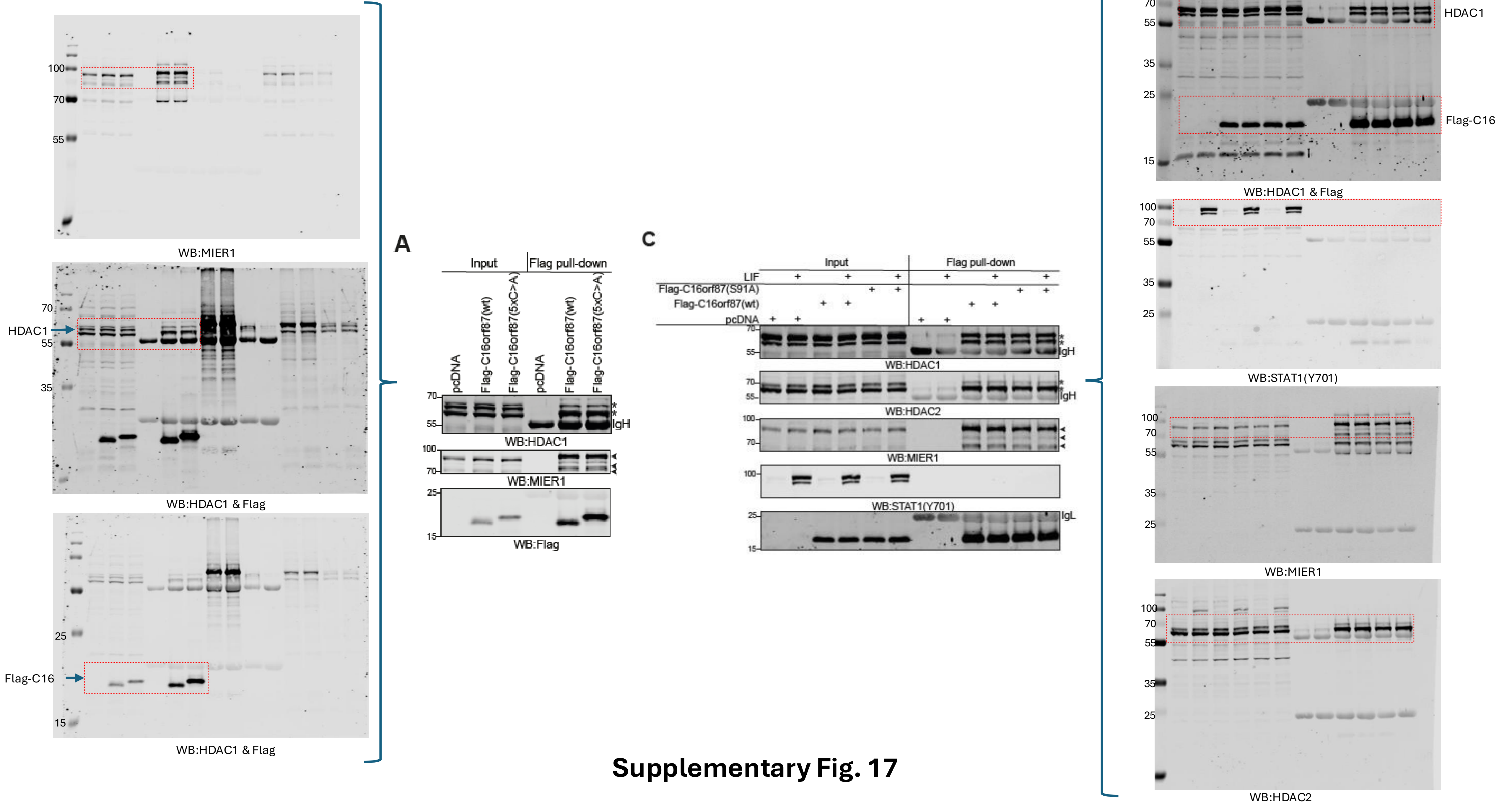

Supplementary Fig. 17

Supplementary Table 2

Cluster	C16orf87 (residue)	Average pLDDT C16orf87	HDAC1 (residue)	Average pLDDT HDAC1	PAE residue-to-residue (Å)	Min. distance (Å)	Catalytic pocket
**Cluster 1** Mean PAE: 20.93Å Mean pLDDT: 60.30	Val-25	49.77	Gln-26	82.05	22.0/22.7	4.291	
	Cys-27	51.46	Glu-98	72.56	18.7/21.3	4.208	X
	Lys-28	45.79	Glu-98	72.56	19.0/22.2	3.869	X
	Ser-29	60.19	Glu-98	72.56	18.7/22.8	2.424	X
**Cluster 2** Mean PAE: 1.56Å Mean pLDDT: 86.81	Glu-142	82.22	Tyr-330	85.80	2.0/1.9	4.127	
	Arg-145	75.27	Tyr-330	85.80	1.9/1.9	4.877	
	Lys-146	88.18	Tyr-330	85.80	1.7/1.5	4.165	
			Asn-331	90.33	1.6/1.4	4.872	
			Asp-332	90.04	1.2/1.2	3.018	
			Tyr-333	92.06	1.5/1.2	4.947	
			Glu-335	83.49	1.8/1.7	3.751	
	Gln-150	84.77	Arg-36	91.70	1.6/1.3	3.590	
			Asn-40	91.06	1.6/1.4	3.609	
			Asp-332	90.04	1.4/1.4	2.957	
			Tyr-333	92.06	1.8/1.3	3.058	
**Cluster 3** Mean PAE: 3.00Å Mean pLDDT: 80.43	Leu-154	72.06	Arg-55	88.79	4.1/1.9	2.796	

Supplementary Table 3

Cluster	C16orf87 (residue)	Average pLDDT C16orf87	MIER1 (residue)	Average pLDDT MIER1	PAE residue-to-residue (Å)	Min. distance (Å)	ELM2-SANT
Cluster 1 Mean PAE: 2.99 Å Mean pLDDT: 80.87	Ile-122	53.27	Lys-301	81.19	10.3/5.3	4.180	S
	Asp-123	61.64	Arg-294	80.54	8.3/4.4	4.974	S
	Ile-124	64.09	Glu-297	88.67	6.3/3.4	4.798	S
			Lys-301	81.19	6.7/3.5	4.169	S
			Phe-338	78.72	5.9/3.9	3.209	
			Phe-339	82.66	7.5/4.2	4.099	
			Gln-342	71.02	5.8/4.9	4.247	
	Tyr-125	66.15	Cys-293	90.60	9.1/3.3	4.760	S
			Arg-294	80.54	5.0/3.0	3.510	S
			Glu-297	88.67	6.1/3.1	1.778	S
			Gln-298	84.21	6.1/3.2	4.848	S
			Arg-335	83.80	8.0/3.5	4.164	S
			Phe-338	78.72	5.5/3.8	4.292	
	Leu-128	68.20	Arg-294	80.54	7.8/3.3	4.239	S
			Phe-338	78.72	6.3/4.0	3.846	
	Lys-132	74.24	Phe-338	78.72	3.2/2.7	4.274	
	Val-135	85.35	Glu-334	78.47	1.8/1.9	3.014	S
	Phe-136	83.21	Glu-290	85.66	1.8/1.7	4.438	S
			Glu-334	78.47	1.8/1.7	3.996	S
			Arg-335	83.80	1.5/1.6	3.273	S
			Phe-338	78.72	2.1/2.4	3.256	
	Ser-137	85.97	Glu-290	85.66	1.8/1.7	4.817	S
			Arg-335	83.80	1.6/2.0	4.564	S
	Ala-139	90.99	Ser-333	87.74	1.3/1.5	4.470	S
			Glu-334	78.47	1.6/1.5	4.454	S
			Arg-335	83.80	1.4/1.5	3.993	S
	Leu-140	89.88	Trp-288	91.11	1.3/1.2	3.728	S
			Thr-289	87.93	1.4/1.3	3.664	S
			Glu-290	85.66	1.3/1.2	3.585	S
			Cys-293	90.60	1.2/1.2	4.060	S
			Phe-326	93.75	1.4/1.2	3.933	S
			Arg-335	83.80	1.4/1.9	4.142	S
	Ile-143	91.21	Phe-326	93.75	1.1/1.0	3.594	S
			Met-329	88.60	1.1/1.2	3.788	S
			Trp-330	91.46	1.2/1.2	3.852	S
			Ser-333	87.74	1.2/1.6	4.130	S
	Asn-144	88.23	Val-287	81.25	1.4/2.2	3.592	S
			Trp-288	91.11	1.0/1.1	2.707	S
	Lys-146	88.18	Met-329	88.60	1.2/1.4	3.661	S
	Ile-147	87.29	Trp-288	91.11	1.3/1.5	4.052	S
			Phe-326	93.75	1.3/1.2	3.955	S
			Met-329	88.60	1.3/1.4	3.431	S
	Ile-148	81.18	Ala-281	62.23	4.9/8.6	4.725	
			Leu-285	67.47	3.0/5.3	4.008	S
			Val-287	81.25	1.8/3.0	3.622	S
	Gln-150	84.77	Glu-254	88.60	1.6/1.7	4.521	E
			Met-329	88.60	1.6/1.6	3.477	S
	Arg-151	69.92	Gln-255	86.48	1.7/1.7	4.229	E
			Leu-285	67.47	3.4/6.1	3.536	S
	Leu-152	82.12	Leu-213	93.61	2.2/1.5	3.748	E
			Glu-254	88.60	2.0/1,5	3.981	E
			Gln-255	86.48	1.6/1.4	3.440	E
			Tyr-258	91.06	2.0/1.4	4.169	E
	Ile-153	76.64	Gln-255	86.48	1.8/1.5	2.665	E
			Tyr-258	91.06	2.1/1.5	3.470	E
			Glu-259	86.31	2.4/1.7	4.797	E
			Lys-262	84.82	2.9/1.9	3.230	E
			Asn-277	61.70	5.6/7.9	3.586	E
			Val-278	61.19	5.2/7.9	4.956	E
			Ala-281	62.23	5.4/7.7	3.475	
	Leu-154	72.06	Gln-211	88.27	4.0/2.0	3.614	E
			Leu-213	93.61	4.0/1.9	3.714	E
			Tyr-258	91.06	3.4/1.7	3.236	E
			Lys-262	84.82	5.2/2.1	3.707	E

Supplementary Table 4

Cluster	HDAC1 (residue)	Average pLDDT HDAC1	MIER1 (residue)	Average pLDDT MIER1	PAE residue-to-residue (Å)	Min. distance (Å)	ELM2-SANT
**Cluster 1**<br>Mean PAE: 2.09 Å<br>Mean pLDDT: 87.75	Lys-10	83.77	Glu-217	79.78	2.2/2.1	2.785	E
	Tyr-14	95.37	Asp-210	90.57	1.2/1.5	4.281	E
	Tyr-15	95.57	Leu-213	93.61	1.1/1.3	4.720	E
			Glu-254	88.60	1.4/1.7	4.688	E
	Gly-20	93.28	Val-324	94.36	1.3/1.2	3.830	S
			Tyr-328	92.63	1.8/1.4	4.725	S
	Asn-21	89.74	Val-320	92.05	1.5/1.2	4.694	S
			Gly-321	94.05	1.2/1.2	3.534	S
			Glu-322	89.91	1.6/1.1	4.884	S
			Val-324	94.36	1.2/1.1	3.389	S
			Ala-325	94.49	1.4/1.2	3.613	S
	Tyr-22	92.78	Val-324	94.36	1.1/1.2	4.282	S
	Tyr-23	92.34	Lys-305	86.34	1.4/1.5	3.325	S
			Phe-307	91.17	1.2/1.2	3.403	S
			Val-324	94.36	1.1/1.1	3.529	S
			Tyr-328	92.63	1.4/1.2	3.700	S
	Gln-26	82.05	Tyr-303	87.02	2.1/1.5	4.479	S
			Gly-304	89.75	1.7/1.5	3.751	S
			Lys-305	86.34	1.5/1.3	2.898	S
			Asp-306	89.86	1.7/1.3	4.442	S
	Lys-31	88.96	Asp-35	52.40	6.1/11.6	2.450	
			Glu-36	55.48	6.3/10.2	3.735	
			Tyr-328	92.63	1.4/1.3	3.819	S
	His-33	91.29	Tyr-328	92.63	1.1/1.1	3.412	S
	Arg-36	91.70	Ala-325	94.49	1.2/1.3	3.392	S
	His-39	94.18	Glu-254	88.60	1.2/1.6	3.549	E
	Leu-42	94.48	Asp-252	89.24	1.1/1.8	4.018	E
	Leu-43	93.58	Ile-250	77.17	1.7/2.9	4.961	E
			Lys-251	78.29	1.4/2.0	3.885	E
			Asp-252	89.24	1.0/1.3	2.951	E
			Glu-254	88.60	1.2/1.3	4.201	E
	Asn-44	90.90	His-249	61.52	3.0/8.5	3.783	E
			Lys-251	78.29	1.4/2.1	3.025	E
			Asp-252	89.24	1.1/1.4	4.865	E
	Tyr-45	94.50	His-249	61.52	2.8/9.5	2.307	E
	Gly-46	93.74	His-249	61.52	3.0/6.5	3.849	E
			Ile-250	77.17	1.9/2.5	4.335	E
			Asp-252	89.24	1.2/1.3	2.952	E
	Leu-47	94.31	Asp-252	89.24	1.1/1.9	3.458	E
	Tyr-48	94.82	Leu-213	93.61	1.2/1.3	3.930	E
			Trp-214	93.67	1.1/1.2	3.911	E
			Asp-252	89.24	1.1/1.4	3.252	E
			Glu-254	88.60	1.2/1.4	3.155	E
			Leu-257	92.65	1.1/1.2	3.517	E
	Arg-49	89.93	Trp-214	93.67	1.2/1.2	2.867	E
			Pro-216	91.93	1.2/1.7	3.410	E
			Glu-221	84.62	1.5/1.6	3.663	E
			Val-224	91.32	1.4/1.2	4.356	E
			Ile-225	88.96	1.5/1.2	4.171	E
			Leu-228	90.18	1.4/1.3	4.058	E
			His-249	61.52	3.1/8.2	4.462	E
			Ile-250	77.17	1.9/3.5	2.789	E
			Lys-251	78.29	1.8/1.9	4.471	E
			Asp-252	89.24	1.1/1.4	2.663	E
			Leu-257	92.65	1.2/1.2	4.114	E
	Lys-50	91.20	Trp-214	93.67	1.1/1.3	4.530	E
			Pro-216	91.93	1.1/1.6	3.879	E
			Glu-221	84.62	1.5/1.8	2.499	E
	Met-51	94.24	Trp-214	93.67	1.1/1.2	3.106	E
			Asp-215	92.25	1.2/1.4	4.831	E
			Pro-216	91.93	1.1/1.5	3.318	E
	Glu-52	92.89	Leu-212	93.54	1.1/1.1	4.042	E
			Trp-214	93.67	1.0/1.0	3.493	E
			Asp-215	92.25	1.1/1.2	4.428	E
			Pro-216	91.93	1.1/1.3	3.372	E
	Ile-53	94.43	Gln-211	88.27	1.1/1.2	4.116	E
			Leu-212	93.54	1.0/1.0	3.633	E
			Leu-213	93.61	0.9/0.9	2.867	E
			Trp-214	93.67	1.0/1.0	2.858	E
			Asp-215	92.25	1.1/1.2	4.990	E
	Tyr-54	94.47	Asp-210	90.57	1.1/1.2	3.928	E
			Gln-211	88.27	1.0/1.0	3.315	E
			Leu-212	93.54	0.9/0.9	3.614	E
			Leu-213	93.61	0.9/0.9	4.344	E

	Arg-55	88.79	Asp-209	83.75	1.4/1.6	4.861	E
			Asp-210	90.57	1.0/1.2	3.483	E
			Gln-211	88.27	1.0/1.0	2.862	E
			Leu-213	93.61	1.0/1.1	3.841	E
	Pro-56	95.63	Asp-209	83.75	1.6/1.8	4.975	E
			Asp-210	90.57	1.2/1.3	3.720	E
	His-57	91.31	Tyr-206	86.33	1.5/1.8	3.413	E
			Asn-208	83.32	1.4/1.4	3.398	E
			Asp-209	83.75	1.5/1.4	3.767	E
			Asp-210	90.57	1.1/1.1	2.722	E
	Lys-58	89.07	Asn-208	83.32	1.4/2.1	3.020	E
	Asn-60	88.87	Glu-207	76.47	1.9/2.1	3.005	E
			Asn-208	83.32	1.7/2.0	4.833	E
	Glu-63	90.78	Tyr-206	86.33	1.5/1.9	3.330	E
			Glu-207	76.47	1.8/2.0	4.749	E
	Lys-66	86.25	Glu-191	81.99	1.3/1.7	4.908	E
			Val-205	83.24	2.0/3.0	4.215	E
	Tyr-67	94.64	Phe-188	89.35	1.3/1.4	3.560	E
			Gln-189	89.81	1.3/1.3	4.243	E
			Ala-190	91.41	1.2/1.3	3.694	E
	Pro-81	89.73	His-308	87.29	1.7/1.5	4.274	S
	Asp-82	82.82	His-308	87.29	1.9/1.8	4.992	S
	Asp-104	87.39	His-308	87.29	1.4/1.3	2.868	S
			Val-320	92.05	1.4/1.3	3.896	S
	Gly-115	96.68	Asp-210	90.57	1.3/1.6	4.733	E
	Val-118	95.42	Tyr-206	86.33	1.5/2.1	4.099	E
	Ala-119	96.12	Tyr-206	86.33	1.4/2.0	3.692	E
			Asp-210	90.57	1.2/1.3	3.429	E
	Ala-121	95.97	Tyr-199	84.27	1.5/3.1	4.783	E
	Val-122	94.18	Tyr-199	84.27	1.4/2.2	3.558	E
			Val-205	83.24	1.6/2.1	3.803	E
			Tyr-206	86.33	1.4/1.9	3.730	E
	Asn-125	92.63	Tyr-199	84.27	1.4/2.0	2.804	E
	Lys-126	89.36	Tyr-199	84.27	1.5/2.0	4.015	E
			Glu-203	75.55	2.2/4.2	4.890	E
			Val-205	83.24	1.7/2.8	4.717	E
	Lys-144	92.03	Ser-186	86.57	1.6/1.5	3.109	E
			Met-187	82.76	1.4/1.4	3.401	E
			Phe-188	89.35	1.2/1.2	3.868	E
			Gln-189	89.81	1.4/1.2	2.910	E
			Ala-190	91.41	1.4/1.3	4.716	E
			Glu-191	81.99	1.6/1.5	4.540	E
	Ser-145	92.22	Met-187	82.76	1.7/1.7	4.309	E
	Val-157	96.17	Ala-190	91.41	1.1/1.4	4.177	E
	Leu-161	94.58	Ala-190	91.41	1.1/1.4	4.147	E
			Glu-191	81.99	1.3/1.4	3.618	E
			Ile-192	87.60	1.2/1.7	4.325	E
			Pro-193	87.29	1.6/2.2	4.131	E
	Glu-162	92.37	Tyr-199	84.27	1.6/2.5	2.729	E
			Val-205	83.24	1.7/2.4	3.108	E
	Leu-164	93.69	Ile-192	87.60	1.3/1.7	4.298	E
			Pro-193	87.29	1.5/2.1	3.838	E
			Gly-195	84.58	1.5/2.7	3.924	E
			Ile-196	79.70	1.3/2.2	4.432	E
	Lys-165	90.48	Pro-193	87.29	1.7/2.8	4.802	E
			Gly-195	84.58	1.6/2.5	4.801	E
			Ile-196	79.70	1.2/1.9	2.976	E
			Cys-197	81.68	1.5/1.9	2.791	E
			Arg-198	73.85	1.7/2.0	3.985	E
			Tyr-199	84.27	1.4/1.8	3.873	E
			Val-205	83.24	1.8/3.1	4.124	E
	Tyr-166	91.03	Ile-196	79.70	1.3/2.0	3.745	E
			Cys-197	81.68	1.5/2.0	4.248	E
			Arg-198	73.85	1.6/2.1	3.705	E
			Tyr-199	84.27	1.5/1.8	2.914	E
	His-167	90.95	Ile-196	79.70	1.3/2.5	4.692	E
	Gln-168	85.27	Gly-195	84.58	1.8/1.8	4.927	E
			Ile-196	79.70	1.5/1.5	4.230	E
	His-179	92.61	Ile-182	86.95	1.3/1.3	4.086	E
	Asp-181	92.16	Ile-182	86.95	1.4/1.4	4.062	E
	Glu-184	93.04	Ile-182	86.95	1.1/1.1	3.445	E
	Glu-185	92.36	Glu-181	78.35	1.5/1.7	4.679	E
			Ile-182	86.95	1.1/1.2	3.843	E
			Met-183	87.59	1.2/1.2	3.251	E
			Phe-188	89.35	1.1/1.2	3.812	E
			Gln-189	89.81	1.0/1.2	3.022	E
	Ala-186	95.18	Phe-188	89.35	1.1/1.2	4.069	E

			Gln-189	89.81	1.0/1.1	3.547	E
			Ala-190	91.41	1.0/1.2	2.893	E
			Ile-192	87.60	1.3/1.8	4.584	E
	Phe-187	94.58	Gln-189	89.81	1.0/1.3	4.968	E
			Ala-190	91.41	1.1/1.3	4.235	E
			Ile-192	87.60	1.3/1.8	3.463	E
	Tyr-188	93.30	Ile-182	86.95	1.1/1.3	3.648	E
			Val-184	88.10	1.2/1.4	3.784	E
			Gln-189	89.81	1.1/1.3	4.177	E
	Thr-189	90.68	Val-184	88.10	1.2/1.4	3.856	E
			Gln-189	89.81	1.1/1.3	3.493	E
	Thr-190	91.55	Ile-192	87.60	1.4/1.7	3.927	E
	Arg-192	90.98	Ile-192	87.60	1.4/1.7	4.034	E
			Pro-193	87.29	1.9/2.2	2.707	E
			Val-194	80.69	2.1/3.7	4.339	E
			Gly-195	84.58	1.9/3.1	3.778	E
			Ile-196	79.70	1.8/2.3	4.703	E
	Tyr-201	90.05	Trp-178	76.53	2.0/3.2	3.781	
			Lys-179	76.12	1.8/2.8	4.277	
	Gly-202	87.20	Trp-178	76.53	2.5/3.0	3.445	
	Glu-203	77.93	Trp-178	76.53	2.0/2.8	3.468	
	Tyr-204	87.30	Trp-178	76.53	1.7/4.1	4.487	
	Pro-206	88.79	Trp-178	76.53	1.9/3.7	4.546	
			Lys-180	80.06	1.4/2.0	3.435	E
	Gly-207	90.03	Trp-178	76.53	1.8/1.9	3.660	
			Lys-179	76.12	1.5/1.7	3.648	
			Lys-180	80.06	1.3/1.4	3.077	E
	Thr-208	89.45	Lys-180	80.06	1.3/1.5	3.414	E
			Glu-181	78.35	1.6/1.5	3.979	E
			Ile-182	86.95	1.4/1.3	3.992	E
	Arg-212	78.75	Lys-179	76.12	2.1/3.9	4.931	
	Asp-213	89.17	Ile-182	86.95	1.5/1.5	4.868	E
	Ala-216	91.75	Val-184	88.10	1.4/1.5	4.271	E
	Gly-268	91.77	Thr-38	57.41	9.8/9.5	3.348	
	Arg-270	78.68	Asp-35	52.40	5.2/9.0	3.000	
			Glu-36	55.48	5.2/7.6	4.711	
			Arg-37	52.24	6.3/5.7	3.732	
	Tyr-303	90.91	Asp-35	52.40	5.7/11.9	4.154	
	Thr-304	91.24	Glu-36	55.48	6.2/8.5	4.183	
			Thr-38	57.41	8.6/8.0	4.026	
	Ile-305	89.29	Glu-36	55.48	5.5/10.4	3.684	
			Tyr-328	92.63	1.5/1.4	4.831	S
	Arg-306	87.09	Glu-36	55.48	5.8/9.1	3.027	
			Arg-37	52.24	6.5/8.1	4.247	
			Thr-38	57.41	9.3/8.1	3.333	
			Glu-41	53.79	9.1/9.1	2.730	
	Asp-332	90.04	Lys-251	78.29	1.8/3.3	4.524	E
	Tyr-333	92.06	Tyr-328	92.63	1.3/1.2	4.538	S
			Met-329	88.60	1.2/1.4	3.574	S
	Glu-335	83.49	Lys-332	85.53	1.3/1.4	3.873	S
	Tyr-336	91.14	Glu-36	55.48	6.2/10.5	2.838	
			Tyr-327	92.75	1.5/1.2	4.816	S
			Tyr-328	92.63	1.3/1.3	2.411	S
			Met-329	88.60	1.4/1.4	4.525	S
			Lys-331	85.81	1.4/1.7	3.983	S
			Lys-332	85.53	1.3/1.5	3.232	S

Supplementary Table 5

Interface 1 (C16orf87 – HDAC1) piCS: 0.94

Link	C16orf87	HDAC1
L1	146	332
L2	150-152	17
L3	150-152	332

Interface 2 (C16orf87 – MIER1) piCS: 0.89

Link	C16orf87	MIER1
L1	124-125	278-294
L2	124-125	342
L3	135-154	211-213
L4	135-154	254-258
L5	135-154	278-294
L6	135-154	325-338

Interface 3 (HDAC1 – MIER1) piCS: 0.95

Link	HDAC1	MIER1
L1	14	205-217
L2	20-27	304-308
L3	20-27	320-332
L4	33	320-332
L5	42-63	205-217
L6	42-63	221-224
L7	42-63	228
L8	42-63	250-257
L9	67	178-199
L10	81	304-308
L11	104	304-308
L12	104	320-332
L13	118-126	178-199
L14	118-126	205-217
L15	143-145	178-199
L16	157	178-199
L17	161-168	178-199
L18	181-192	178-199
L19	202-208	178-199
L20	213-216	178-199
L21	329-336	250-257
L22	329-336	320-332
