# Supplementary material for: The C16orf87 protein is a subunit of the MIER corepressor complex controlling embryonic development and cell migration
Source: Sci Rep. 2026 Apr 30;16:13907. doi: 10.1038/s41598-026-50740-7 (PMC13133221; doi:10.1038/s41598-026-50740-7)
Supplement: Supplementary file 5 — Supplementary Information 4. [file 41598_2026_50740_MOESM5_ESM.docx]

**Supplementary Figure 1. Structural confidence and coiled-coil prediction for C16orf87.** (**A**) A schematic drawing of the human C16orf87 (Uniprot Q6PH81). A putative zinc ribbon domain (ZRD, Pfam domain UPF0547), individual cysteines (Cys; C16, C19, C27, C30, and C32) within the ZRD, and a dominant phosphorylation site (S91) are indicated. (**B**) Predicted Aligned Error (PAE) plot from AlphaFold3 of the predicted C16orf87 model. Low PAE values along the main diagonal indicate confident intra-domain modeling, while higher values off the diagonal reflect uncertainty in relative domain orientation. (**C**) Coiled-coil predictions for C16orf87 obtained with six independent tools: NCOILS, PairCoil2, DeepCoil, Marcoil, DeepCoil2, and CoCoNat. Each plot shows the coiled-coil probability as a function of the residue position. All plots are from the MPI Bioinformatics Toolkit, except the NCOILS and CoCoNat plots, as Waggawagga only shows plots where coiled-coils are predicted (no plots are shown from MultiCoil, MultiCoil2 or PairCoil). The SAH windows score in the NCOILS plot indicates the likelihood of a single alpha-helix (SAH) region. CoCoNat also shows oligomerization state and hepad repeat registers if coiled-coils are predicted.

**Supplementary Figure 2. CRISPR-Cas9 genome editing of the C16orf87 locus in Panc-01 cells.** An illustration of the Panc-01^KO^ clone, lacking 158 nucleotides at the exon 1/intron 1 boundary. The excision site is marked by a purple arrow, and a blue box shows the deleted sequence ("Del in Panc01 2A6").

**Supplementary Figure 3. C16orf87 putative zinc-ribbon domain and Ser91 phosphorylation are not needed for HDAC1 and MIER1 interactions.** (**A)** Five-point mutations within the C16orf87 zinc-ribbon domain (ZRD; Cys16Ala, Cys19Ala, Cys27Ala, Cys30Ala, and Cys32Ala; collectively as 5xC>A) do not alter C16orf87 interaction with the HDAC1 and MIER1 proteins. HeLa cells were transfected with the indicated plasmids, followed by Flag antibody-coupled magnetic beads, and isolation of the C16orf87-interacting proteins. Proteins were detected with Western blot (WB) analysis. (**B)** Volcano plot of phosphopeptides from the phosphoproteomics experiment of C2C12 cells treated with mouse Leukemia Inhibitory Factor (LIF; 100 nM, 15 min). Phosphorylated C16orf87 is indicated, and the pink box highlights statistically significant data points (FDR: 0.05, fold-change: 1.5, n=3). (**C)** LIF treatment does not alter the interaction of C16orf87 with HDAC1, HDAC2, and MIER1. HeLa cells were transfected with Flag-C16orf87 and a potential Ser91 phosphorylation-deficient mutant Flag-C16orf87(S91A) expressing plasmids. Cells were also treated with human LIF to activate the intracellular Akt/PKB phosphorylation pathway. Flag-C16orf87 interacting proteins were isolated with anti-Flag antibody-coupled magnetic beads, and the interacting proteins were analyzed using Western blotting (WB) with the indicated antibodies. Detection of STAT1 phosphorylated residue Tyr-701 serves as a positive control for a LIF-induced phosphorylation cascade. (**D)** Purified, His-tagged C16orf87 does not block HDAC1 enzymatic activity using an acetylated peptide as a substrate. Irrelevant, His-tagged purified protein (His-MS2) was used as the specificity control. TSA, a known inhibitor of the HDACs, was used to validate HDAC1 enzymatic activity. Average of 3 independent measurements, data shown as the relative HDAC1 activity after considering the sample without adding HDAC1 as 1.

**Supplementary Figure 4. Predicted structure and confidence metrics of a possible dimeric C16orf87.** (**A**) Cartoon representation of the top-ranked predicted dimeric complex of C16orf87 colored by per-residue confidence (pLDDT). Interface predicted TM-score (ipTM), predicted TM-score (pTM), actifpTM, and ipSAE values for the top-ranked model are shown. N, N-terminus; C, C-terminus. (**B**). Predicted Aligned Error (PAE) plot from AlphaFold-Multimer of the predicted dimeric model, showing high uncertainty.

**Supplementary Figure 5. Purified, His-tagged C16orf87 does not block HDAC1 enzymatic activity using an acetylated peptide as a substrate.** Irrelevant, His-tagged purified protein (His-MS2) was used as the specificity control. TSA, a known inhibitor of the HDACs, was used to validate HDAC1 enzymatic activity. Average of 3 independent measurements, data shown as the relative HDAC1 activity after considering the sample without adding HDAC1 as 1.

**Supplementary Figure 6. Predicted structure and confidence metrics of the C16orf87–HDAC1–MIER1 complex from AlphaFold3.** (**A-C**) Individual proteins extracted from the predicted C16orf87–HDAC1–MIER1 complex are shown as cartoon models colored by per-residue confidence (pLDDT). (A) HDAC1, (B) MIER1 and (C) C16orf87. (**D**) HDAC1 (peach) and MIER1 (lavender grey) from the C16orf87–HDAC1–MIER1 complex are shown as a cartoon model. The ELM2 domain (blue) and SANT domain (red) from MIER1 are highlighted, with residue numbers indicating their domain boundaries. (**E**) C16orf87 (pastel green) and MIER1 (lavender grey) from the C16orf87–HDAC1–MIER1 complex are shown as a cartoon model. The ELM2 domain (blue) and SANT domain (red) from MIER1 are highlighted, with residue numbers indicating their domain boundaries. (**F**) Predicted Aligned Error (PAE) plot from AlphaFold3 of the predicted heterotrimeric model, showing low intra-domain error for the HDAC1 and the ELM2-SANT region of MIER1, but higher uncertainty for the flexible N- and C-terminal regions of MIER1 and C16orf87.

**Supplementary Figure 7. Contact clustering analysis of the C16orf87–HDAC1–MIER1 complex.** (**A**) Clusters of the C16orf87–HDAC1 (blue), C16orf87–MIER1 (orange), and HDAC1–MIER1 (green) interfaces determined by PICKLUSTER are highlighted on the model of the C16orf87–HDAC1–MIER1 complex from AlphaFold3. (**B**) Predicted Merged Confidence (PMC) matrix from AlphaBridge of the C16orf87–HDAC1–MIER1 complex model, as generated by AlphaFold3, used to define the binary interfaces. (**C**) Predicted Distance Error (PDE) matrix of the C16orf87–HDAC1–MIER1 complex model from AlphaFold3, showing the confidence that two residues are in contact (<8 Å). The PDE is used in AlphaBridge to help define the binary interfaces. (**D**) Chord diagram from AlphaBridge summarizing the predicted inter-chain residue-residue contacts within the whole complex. The outer ring represents the different proteins in the complex with the residue numbers annotated. The functional regions ELM2 (blue) and SANT (red) from MIER1, and the zinc-ribbon domain (ZRD, violet) and alpha-helix (light blue) from C16orf87 are annotated. The inner ring represents the model confidence (local pLDDT) of the proteins, colored as in Fig. 5b. Interfaces between the proteins are depicted as curves ("bridges") between regions that make up the contact links. Bridges between C16orf87 and HDAC1 are in blue, C16orf87 and MIER1 in orange, and HDAC1 and MIER1 in green. (**E**) Clusters of the C16orf87–HDAC1 (blue), C16orf87–MIER1 (orange), or HDAC1–MIER1 (green) interfaces determined by AlphaBridge are highlighted on the model of the C16orf87–HDAC1–MIER1 complex from AlphaFold3. Predicted interaction confidence scores (piCS) are annotated.

**Supplementary Figure 8. Structural superposition of the AlphaFold3 model and the HDAC1–MTA1 X-ray structure.** The AlphaFold3 model of the C16orf87–HDAC1–MIER1 complex was structurally aligned with the X-ray structure of HDAC1 (maroon) bound to the ELM2-SANT domain of MTA1 (yellow; PDB 4BKX ^35^). C16orf87 in pastel green, HDAC1 in peach, and MIER1 in lavender grey. The Root-Mean-Square Deviation (RMSD) between the aligned structures is annotated.

**Supplementary Figure 9. C16orf87 alters chromatin accessibility.** (**A**) Gene locus diagrams showing genomic regions altered in Panc-01^WT^ (blue) and Panc-01^KO^ (green) cells. The peaks represent ATAC-seq reads corresponding to chromatin accessibility. The red boxes show loci with statistically significant changes (*p*-adjusted value ≤0.05). HDAC1, HDAC2, MIER2, MIER3, and modified histone tracks are shown below the reference gene track. **(B)** Average signal profiles and heatmap of the peaks classified by higher (left) and lower (right) chromatin accessibility in the Panc-01^KO^ cell line.

**Supplementary Figure 10.** (**A**) qRT-PCR analysis of the *NCOA7*, *HINT3*, *WWOX*, and *C16orf87* mRNA expression in Panc-01 Wt and three knockout clones (A6, H5, and H9). Data shown after normalization to the HRPT1 and considering mRNA levels in Panc-01^Wt^ cells as 1. (**B**) WB analysis of the clones from panel B. Whole cell lysates were analyzed for C16orf87 and tubulin (as a loading control). Relative intensity of the C16orf87 protein after normalization to the tubulin is shown. (**C**) qRT-PCR analysis of the *NCOA7*, *HINT3*, *WWOX*, and *C16orf87* mRNA expression. Relative mRNA expression in siScr- and siC16-treated HeLa cells at 48 h post-transfection. Data shown after normalization to the 18S rRNA and considering mRNA levels in siScr-treated HeLa cells as 1.

**Supplementary Figure 11.** Full-length and uncropped Western blot images corresponding to Fig. 1c.

**Supplementary Figure 12.** Full-length and uncropped Western blot images corresponding to Fig. 2a.

**Supplementary Figure 13.** Full-length and uncropped Western blot images corresponding to Fig. 4b.

**Supplementary Figure 14.** Full-length and uncropped Western blot images corresponding to Fig. 4c.

**Supplementary Figure 15.** Full-length and uncropped Western blot images corresponding to Fig. 4d.

**Supplementary Figure 16.** Full-length and uncropped Western blot images corresponding to Supplementary Fig. 10b.

**Supplementary Figure 17.** Full-length and uncropped Western blot images corresponding to Supplementary Fig. 3a,c.

**Supplementary Table 1. Quantitative MS-analysis of protein abundance in Panc-01^KO^ and Panc-01^WT^ cells.** Quantitative differential analysis of protein abundance in Panc-01^KO^ versus Panc-01^WT^ cells. The analysis of the data generated by LC-MS/MS was performed using Proteome Discoverer 2.5 (Thermo Fisher Scientific), using the human proteome (UP000005640) as reference.

**Supplementary Table 2. The three clusters of the C16orf87-HDAC1 interface as identified by PICKLUSTER.** Mean predicted aligned error (PAE) and predicted local distance difference test (pLDDT) values are reported for each cluster. Interacting residues and their average pLDDT (mean of all included atoms) are listed. Predicted Aligned Error (PAE) for each interaction is shown in both directions (residue A to residue B, and residue B to residue A). The distance between the closest atoms in each interaction (Min. distance) is listed. Residues that contribute to the catalytic pocket of HDAC1 are marked with X.

**Supplementary Table 3. The cluster of the C16orf87–MIER1 interface as determined by PICKLUSTER.** Mean predicted aligned error (PAE) and predicted local distance difference test (pLDDT) values are reported for the cluster. Interacting residues and their average pLDDT (mean of all included atoms) are listed. Predicted Aligned Error (PAE) for each interaction is shown in both directions (residue A to residue B, and residue B to residue A). The distance between the closest atoms in each interaction (Min. distance) is listed. Residues belonging to the ELM2 or SANT domains of MIER1 are labeled E or S, respectively.

**Supplementary Table 4. The cluster of the HDAC1-MIER1 interface as determined by PICKLUSTER.** Mean predicted aligned error (PAE) and predicted local distance difference test (pLDDT) values are reported for the cluster. Interacting residues and their average pLDDT (mean of all included atoms) are listed. Predicted Aligned Error (PAE) for each interaction is shown in both directions (residue A to residue B, and residue B to residue A). The distance between the closest atoms in each interaction (Min. distance) is listed. Residues belonging to the ELM2 or SANT domains of MIER1 are labeled E or S, respectively.

**Supplementary Table 5. Interfaces of the C16orf86–HDAC1–MIER1 complex as determined by AlphaBridge.** Each of the contact links that make up the interface is listed with residue numbers of each protein. piCS values of each interface are also annotated.

**Supplementary Table 6.** **Quantitative ATAC-seq data analysis of loci changed in Panc-01^KO^ and Panc-01^WT^ cells.** Sequences were aligned to the human genome (GRCh38) and analysed by the NF-core ATAC-seq pipeline. The resulting quantitative values were compared using DESEQ.

**Supplementary Video 1. AlphaFold3 predicted model of the C16orf87–HDAC1–MIER1 complex.** C16orf87 (pastel green), HDAC1 (peach), and MIER1 (lavender grey) are shown, with HDAC1 and MIER1 rendered as surface representations and C16orf87 displayed as a cartoon representation.
